# Leaping into the Unknown World of *Sporisorium scitamineum* Candidate Effectors

**DOI:** 10.3390/jof6040339

**Published:** 2020-12-04

**Authors:** Natália Sousa Teixeira-Silva, Patrícia Dayane Carvalho Schaker, Hugo Vianna Silva Rody, Thiago Maia, Christopher M. Garner, Walter Gassmann, Claudia Barros Monteiro-Vitorello

**Affiliations:** 1 Departamento de Genética, Escola Superior de Agricultura “Luiz de Queiroz”, Universidade de São Paulo, Piracicaba CEP 13418-900, Brazil; nataliasteixeirasilva@gmail.com (N.S.T.-S.); patriciaschaker@utfpr.edu.br (P.D.C.S.); hugorody@gmail.com (H.V.S.R.); thiago.maia@usp.br (T.M.); 2 Christopher S. Bond Life Sciences Center and Interdisciplinary Plant Group, University of Missouri, Columbia, MO 65211, USA; cmgarner07@gmail.com; 3 Division of Plant Sciences, College of Agriculture, Food and Natural Resources, University of Missouri, Columbia, MO 65211, USA

**Keywords:** effector proteins, subcellular location, fungal effector, sugarcane smut, plant immunity, agroinfiltration, *Nicotiana benthamiana*

## Abstract

*Sporisorium scitamineum* is a biotrophic fungus causing sugarcane smut disease. In this study, we set up a pipeline and used genomic and dual transcriptomic data previously obtained by our group to identify candidate effectors of *S. scitamineum* and their expression profiles in infected smut-resistant and susceptible sugarcane plants. The expression profile of different genes after infection in contrasting sugarcane genotypes assessed by RT-qPCR depended on the plant genotypes and disease progression. Three candidate effector genes expressed earlier only in resistant plants, four expressed in both genotypes, and three later in susceptible plants. Ten genes were cloned and transiently expressed in *N. benthamiana* leaves to determine their subcellular location, while four localized in more than one compartment. Two candidates, g3890 having a nucleoplasmic and mitochondrial location and g5159 targeting the plant cell wall, were selected to obtain their possible corresponding host targets using co-immunoprecipitation (CoIP) experiments and mass spectrometry. Various potential interactors were identified, including subunits of the protein phosphatase 2A and an endochitinase. We investigated the presence of orthologs in sugarcane and using transcriptome data present their expression profiles. Orthologs of sugarcane shared around 70% similarity. Identifying a set of putative fungal effectors and their plant targets provides a valuable resource for functional characterization of the molecular events leading to smut resistance in sugarcane plants and uncovers further opportunities for investigation.

## 1. Introduction

Despite being considered a robust and highly tolerant crop, sugarcane is affected by various pests and pathogens, some of which drastically compromise productivity. Sugarcane smut, caused by *Sporisorium scitamineum* (Syd.) (Piepenbring et al., (2002) (Syn: *Ustilago scitaminea* H. and P. Sydow)), is among the diseases affecting the crop across the globe [1,2,3]. *S. scitamineum* is a biotrophic fungus specialized in defeating sugarcane defenses to complete its life cycle [4,5]. The pathogen interferes with sugarcane metabolism in such a way that the plant architecture is modified, leading to the production of a very particular whip-like structure from the apex of infected plants [2,3,6].

The fungus has a host-dependent life cycle starting with teliospore germination in bud surfaces at the base of leaf insertions. Germination generates a probasidium where meiosis produces haploid sporidial cells. Mating compatible haploid cells fuse to produce a dikaryotic infective hypha [7]. The penetration of sugarcane tissues involves appressorium formation enabling inter- and intracellular colonization. In advanced stages, the fungus induces sporogenesis by a still-unknown signaling pathway. The life cycle ends with the emergence of the whip-like structure from the shoot apical meristem (SAM), where billions of teliospores produced in a single whip are easily dispersed in the field by wind, rain, and small animals. Disease establishment in young plants results in tillering and narrow leaves. Altogether, these symptoms culminate in decreased biomass and low juice quality [2,8,9].

The mechanisms involved in the interplay between molecules of sugarcane and *S. scitamineum* are still poorly understood. One of the metabolic and physiological orchestrators in plant–pathogen interactions is effector proteins. Effectors produced by host-associated organisms interfere with host resistance or susceptibility, depending on the genetic background [10]. They have evolved to subvert host defenses and promote effector-triggered susceptibility (ETS) [11]. In recent years, many effectors were functionally characterized as acting in remarkably diverse ways. When delivered to the host apoplast, they can act as degrading enzymes and inhibitors of host proteases, interfere in chitin perception, detoxify host environments and block oxidative burst responses [11,12,13,14]. Effectors can also be deployed inside the host cell, and in this case, can target a variety of organelles and biomolecules interfering in several pathways. These proteins can interact with host proteins to avoid immune responses, alter hormone signaling cascades, divert the biosynthetic pathway of secondary metabolites, redirect nutrient acquisition from the host to support their feeding requirements, and interfere with host transcriptional regulation by acting as transcription factors [15,16,17,18,19]. All these mechanisms culminate in improved pathogen fitness against the host immune system.

The increasing availability of plant–pathogen genome sequences and the dual transcriptomic data of host–pathogen interactions, in addition to the advantages of heterologous systems, has effectively contributed to the study of effector biology and its application in plant protection and breeding programs [20,21]. Historically, individual effectors that are recognized by host resistance (R) proteins were termed avirulence (AVR) proteins. Since the early description of AVR)– R protein interactions resulting in plant resistance, more complex interactions between *AVR* and *R* genes were reported, especially considering fungal pathogens [22]. Although some of these elaborate mechanisms were deciphered for various pathosystems, this is not the case for sugarcane–fungal interactions. Sugarcane has a highly polyploid genome, originated from interspecific hybridizations, that impose enormous difficulties in detecting such networks of events [23]. Especially for sugarcane smut, an additional limitation is the lack of specific phenotypes in infected plants at early stages, hindering assays and functional characterizations. The most notorious symptom, the whip, is visible at the earliest at 45 days after inoculation in highly susceptible genotypes, and is often asynchronous and highly dependent on environmental factors. 

A robust and sustainable way to control fungal diseases involves resistant genotypes, which requires a profound understanding of the host–pathogen interaction and years of breeding strategies. Compared to other pathosystems, studies regarding sugarcane smut resistance fall behind in fungal determinants of virulence. Although effector biology is a new theme of investigation in the sugarcane–*S. scitamineum* pathosystem, effectors in general have been known, for some time, as critical molecules to defeat plant defense mechanisms. This article will discuss the topic of *S. scitamineum* effector biology by addressing selected candidates for gene expression, fluorescently-tagged localization in *N. benthamiana* leaf cells, the impact on plant immunity, and identification of potential interactors using co-immunoprecipitation (CoIP) followed by liquid chromatography–tandem mass spectrometry (CoIP/LC-MS/MS). The set of candidates suggests that the fungus targets various processes in different subcellular compartments, and that the expression of effectors is influenced by the sugarcane genotypes during tissue colonization. Two potential host interactors, an endochitinase and a protein phosphatase 2A, are promising candidates for further investigations.

## 2. Materials and Methods

### 2.1. Ethics Statement and Material Collection

The *S. scitamineum* SSC39 teliospores and derivatives SSC39A and SSC39B haploid cells were obtained from infected plants collected in the experimental field of the IAC sugarcane breeding program (Instituto Agronômico, Centro de Cana, Ribeirão Preto, São Paulo, Brazil) as previously described by Taniguti et al. [4]. We used healthy buds from the sugarcane genotypes clone IAC66-6 (highly susceptible) and the variety SP80-3280 (resistant) obtained from the IAC nursery. The project, conducted in collaboration with IAC, did not require specific permissions to sample spores and plants and did not involve endangered or protected species.

### 2.2. Biological Material and Experimental Conditions

Sugarcane single bud sets collected from 10-month-old plants were surface-sterilized [24] and incubated in humid chambers at 28 °C overnight for pre-germination. We used teliospores of the *S. scitamineum* SSC39 strain [4] with an estimated germination rate of 90% to inoculate the buds. A total of 5 µL of a teliospore paste (10^9^ teliospores mL^−1^) was drop-inoculated over the buds, according to Peters et al. [25] (Figure S1a–d).

Each sample comprised a bulk of six single bud sets that we collected at 0, 72 hai (hours after inoculation), and 5 dai (days after inoculation). Germinated buds transplanted into 2 L pots filled with the substrate Basaplant and enriched with fertilizer containing nitrogen, phosphate, and potassium (NPK) at the ratio 5:25:25 were cultivated under greenhouse conditions with controlled irrigation and a completely randomized design (Figure S1e–f). Apical meristematic regions were collected from plants at the same age with and without whips (Figure S2).

*S. scitamineum* cells were grown overnight in liquid YM media (0.3% yeast extract, 0.3% malt extract, 0.5% soybean peptone, 1% d-glucose) at 28 °C under shaking (200 rpm). Cells were pelleted and stored at –80 °C. Compatible mating-type cells (SSC39A and SSC39B) [4] were mixed, immediately plated in YM media covered by a sterile nitrocellulose membrane, and incubated overnight at 28 °C to produce hyphae. Samples were detached from the membrane and stored at –80 °C. We conducted the assays in triplicates. The pathogen was detected and quantified by RT-qPCR and qPCR to confirm successful infection as previously described [25,26].

### 2.3. Selection of Candidate Effectors and Sequence Analysis

We selected candidate effectors (CE) based on the *S. scitamineum* annotated genome [4] and the dual transcriptome analysis of a compatible interaction [4,5] using the following criteria: (1) genes encoding proteins of the predicted secretome based on the programs SignalP V4.0.1c, TMHMM V2.0c, and predGPI previously described [4]; (2) the list of CEs described by Benevenuto el al. [27]; (3) genes differentially expressed during the interaction at 5 dai; (4) genes differentially expressed during the interaction at 200 dai; (5) genes unique to the *S. scitamineum* genome (OrthoMCL cut-off for Singleton, Orthologs, and Paralogs = e-value 1e-5, similarity 50%), and (6) genes expressed only *in planta*. Additionally, the preferentially expressed genes, i.e., those having an elevated number of mapped reads at a specific time-point (5 or 200 dai), were considered (Figure S3, Table S1). 

The candidates had their sequences analyzed for polymorphisms amongst the four available *S. scitamineum* genome sequences recovered from GenBank (PRJEB6265, PRJNA275631, PRJNA240344, PRJEB5169), derived from Brazil, South Africa, Australia, and China, respectively. The alignments performed using CLC Genomics Workbench V7.01 (CLC Bio, Aarhus, Denmark) were manually inspected for each polymorphic site. The sequence features of the encoded proteins were analyzed using InterPro [28], SignalP [29], ProtParam [30], NLS Mapper [31], RADAR [32], and ApoplastP [33].

### 2.4. Candidate Effector Gene Expression

We designed primers for each predicted Coding-DNA sequence (CDS) and checked their quality and the absence of secondary structures using Primer Blast (https://www.ncbi.nlm.nih.gov/tools/primer-blast/) and NetPrimer (http://www.premierbiosoft.com/netprimer/) (Table S2). Dissociation curves for each target gene confirmed the presence of unique amplicons (Figure S4). The housekeeping gene coding for tubulin-beta chain (*g1237_chr02_Ss*) was used to normalize expression signals.

Total RNA from in vitro cultures and inoculated plants was extracted using TRIzol^®^ Plus RNA Purification Kit (Life Technologies, Carlsbad, CA, USA) according to manufacturer’s specifications and submitted to DNAse treatment using DNAse I (Sigma Aldrich, St. Louis, MO, USA). cDNAs were synthesized with 4 µg of total RNA and Oligo(dT) primers according to the kit GoScript™ Reverse Transcription System (Promega, Madison, WI, USA) instructions. A total of 2 µL of a 10-fold diluted cDNA was used as a starting sample to set up the reactions.

The qPCR reactions were run in a 7500 Fast Real-Time PCR System (Applied Biosystems, Waltham, MA, USA) using the GoTaq^®^ qPCR Master Mix (Promega, Madison, WI, USA). All samples were composed of three biological replicates and two technical replicates. We applied the LinReg PCR program [34] to obtain PCR efficiencies and Cq values, and discarded those Cq values higher than 34 for expression analysis. The relative expression ratios were calculated by the ΔCt method, with *tubulin-beta* as the normalization reference. We applied a one-way ANOVA followed by Tukey’s test (*p* < 0.05) to confirm the observed differences in gene expression. The heatmap was designed using R software.

### 2.5. Vectors and Cloning Procedures for Subcellular Location Assays

Each CE protein had a signal peptide (SP) predicted using SignalP 4.0 [29]. According to the manufacturer’s instructions, primers designed for recombination into the Gateway cloning system included sequences encoding each of the mature proteins without the signal peptide (Table S3).

Amplifications performed with high fidelity polymerase (KAPA HiFi HotStart PCR Kit—Kapa Biosystems, Roche, Basel, Switzerland) were verified for the correct size by gel electrophoresis and column purified (Illustra GFX PCR DNA and Gel Band Purification Kit—GE Healthcare, Chicago, IL, USA) before recombination and cloning. The Table S4 lists all the vectors used in this work. Recombinant vectors were transformed into chemically competent DH10B cells and column-purified from single colonies (GeneJET Plasmid Miniprep Kit—Thermo Fisher Scientific, Waltham, MA, EUA). Constructs were verified for the correct insertions by restriction digestion and sequencing.

### 2.6. Agrobacterium-Mediated Transient Expression and Confocal Microscopy

Expression vectors were electroporated in the GV3101 (pMP90) *A. tumefaciens* strain. Single colonies grown overnight in low salt LB medium (1% Tryptone, 0.5% yeast extract, 0.25% NaCl, pH 7.0) were pelleted and resuspended in infiltration buffer (10 mM MgCl_2_, 10 mM MES, 200 µM acetosyringone) and sat at RT for 4 h. Cells were syringe-infiltrated into 4–5-week-old *Nicotiana benthamiana* leaves at OD_600_ = 0.2. All the constructs were co-infiltrated with pSITE_4NA for free RFP (Red Fluorescent Protein) delivery and nucleus/cytoplasmic localization, and with the silencing suppressor HC-Pro to reduce gene silencing. When necessary, effector candidates were co-infiltrated with cellular markers to confirm localization (Table S4). Protein accumulation in plant cell compartments was verified by confocal laser-scanning microscopy (Leica TCP SP8—Leica Microsystems, Wetzlar, Germany) two days after infiltration. Live-cell imaging performed with a combination of filters allowed the detection of YFP (Yellow Fluorescent Protein) or Citrine, RFP and chlorophyll.

Immunoblots confirmed the presence of the fusion proteins. *N. benthamiana* leaves were harvested, frozen in liquid nitrogen, and ground into a powder with mortar and pestle. Total protein was extracted from 100 mg of tissue powder using 2× SDS Buffer (100 mM Tris pH 6.8, 4% SDS, 20% glycerol, and 100 mM DTT) supplemented with 1× Protease Inhibitor Cocktail (Sigma Aldrich, St. Louis, MO, USA). A total of 30 µL of the isolated proteins was added to 1× Loading Dye/DTT (10% SDS, 20% glycerol, 0.2M Tris pH 6.8, 0.05% bromophenol blue and 250 mM DTT), boiled for 5 min and separated by 12% SDS-PAGE gel. Separated proteins were transferred to a PVDF membrane (Immobilon-P, Millipore, Burlington, MA, EUA) by semi-dry blotting. Blocking occurred in 5% skimmed milk powder in phosphate-buffered saline (137 mM NaCl; 2.7 mM KCl, 10 mM Na2HPO4, 1.8 mM KH2PO4) supplemented with 0.1% Tween 20. YFP and Citrine detections were performed in a two-step fashion using a polyclonal anti-GFP antibody (1:5000, Sigma) and the secondary antibody goat anti-mouse IgG conjugated with Horseradish peroxidase (1:15000, GeneScript, Piscataway, NJ, EUA). Protein fusion bands were visualized by chemiluminescence (ECL™ Prime Western Blotting System—GE Healthcare, Chicago, IL, USA) and revealed in MXBE Kodak film (Carestream, Rochester, NY, USA).

### 2.7. Co-Immunoprecipitation and Mass Spectrometry

*N. benthamiana* leaves were harvested two days after infiltration, frozen in liquid nitrogen, and ground into powder. The empty vector expressing free GFP was used as a negative control. Total protein was extracted from 1 g of tissue powder using 4 volumes of IP Buffer (50 mM Tris pH 7.5, 150 mM NaCl, 25% glycerol, 10 mM EDTA, 10 mM NaF, 10 mM Na_3_VO_4_, 1% Tween 20) supplemented with 1x Protease Inhibitor Cocktail (Sigma Aldrich, St. Louis, MO, USA). Samples were incubated in a rotator shaker at 4 °C for gentle homogenization and centrifuged in ultracentrifuge for 10 min at 40,000× *g* at 4 °C. Supernatants were collected and incubated with 20 µL of anti-GFP beads (Sigma Aldrich, St. Louis, MO, USA) in a rotating shaker at 4 °C for 2 h. Samples were washed three times in Wash Buffer (IP buffer 0.2% Tween 20, no inhibitors) and the residual liquid removed with a nano syringe. Beads were resuspended in 100 µL H_2_O, added to 1× Loading Dye/DTT and boiled for 5 min. Samples were stored at –80 °C.

Immunoblots prepared from a 10% SDS-PAGE gel and transferred to a PVDF membrane (Immobilon-P, Millipore, Burlington, MA, EUA) in semi-dry blotting were used to check the integrity of the fusion proteins and the efficiency of CoIP. A total of 10 µL of the input and immunoprecipitated samples were used. YFP and Citrine detections were performed as described above. Samples were submitted to the Proteomics Core at the University of Missouri for LC-MS/MS analysis in a LTQ Orbitrap XL (Thermo Fisher Scientific, Waltham, MA, EUA).

Raw data were searched using PEAKS X- with the NCBI *Nicotiana tabacum* (76253) database. Data were searched with the following: trypsin as enzyme and 2 missed cleavages allowed; carbamidomethyl cysteine as a fixed modification; oxidized methionine, deamidation of asparagine and glutamine as variable mod; 50 ppm mass tolerance on precursor ions and 0.1 Da on fragment ions. The proteins were identified when the peptide spectrum matched FDR 1% and protein group FDR 0.4% with at least 1 unique peptide per protein. The average spectral counts of each protein were calculated from three replicates and considered significant if an average spectral count exceeded 2. For each CE fusion protein, a score was calculated according to Petre et al. [35]. Immunoprecipitated proteins shared amongst the fusion effectors were discarded. Thus, the final candidate interactors consisted of exclusive proteins for each CE with a Score ≥ 5 and at least 3 unique peptides mapped. The proteins for which multiple subunits or orthologs were found were kept to support process enrichment, even with lower scores.

### 2.8. Determining Sugarcane Orthologs of Nicotiana Interactors

Sugarcane orthologs of *Nicotiana* protein targets predicted to interact with g3890 and g5159 CE during our proteomics analysis were surveyed through a two-step BLASTp search [36]. In the first step, targeted *Nicotiana* sequences were used as queries against a local database of sugarcane largest ORF sequences obtained from a collection of 88,488 sugarcane transcripts [5,37]. Cutoff parameter values to determine a subject hit for each query were set as 1e^−05^, identity ≥ 40%, and query coverage ≥ 80%. Second, a BLASTp search was performed using the sequences from the sugarcane ORFs database as queries against a local database assembled for the targeted *Nicotiana* sequences, still applying the same cutoff parameters.

Python3 scripts were used to parse the BLASTp results and determine ortholog groups by modeling graphs with the help of NetworkX [38]. In the graphs, nodes were the sequences, and edges were the connections among nodes when a query–subject relationship existed. All the predicted connected components were declared as orthogroups among *Nicotiana* and sugarcane.

Finally, we inferred the maximum likelihood phylogenetic relationships among sequences within each orthogroup using the IQTree software [39]. First, multiple sequence alignment was performed using the Muscle software [40] with default parameters. Alignments were inspected manually to avoid sugarcane sequences that could have been derived from transcriptome misassemblies. The best-fit model of molecular evolution for each tree was selected by the ModelFinder software [41] with the parameter -m MFP, by choosing the model that minimizes the Bayesian information criterion (BIC) score. The ultrafast bootstrap approximation (UFBoot) [42] parameter was set as -B 1000 to specify the number of bootstrap replicates, alongside the hill-climbing nearest neighbor interchange (NNI) search with parameter -bnni to reduce the risk of overestimating branch supports. The consensus trees were visualized and edited with FigTree v1.4 (http://tree.bio.ed.ac.uk/, last accessed 08/09/20).

All predicted sugarcane orthologs had their expression profiles determined from two transcriptome experiments performed with sugarcane genotypes harboring divergent degrees of resistance (susceptible and resistant) at 48 hai with smut [43].

*Nicotiana* interactors of CEs having orthologs in sugarcane and showing distinct expression patterns among susceptible and resistant genotypes in the aforementioned transcriptome experiments were further investigated and classified by incorporating *A. thaliana* sequences into the BLASTp orthology searches and phylogenies.

### 2.9. Virulence Assay

For disease induction, the deletion mutant *Pseudomonas syringae* pv. tomato (*Pst*) ΔhopQ1-1 (mutant CUCPB5460) was grown in King’s B plates for 2 to 3 days at 30 °C. Three days after agroinfiltration for transient expression of effectors, *N. benthamiana* leaves were syringe-infiltrated with *Pst* ΔhopQ1-1 suspension at 10^6^ CFU mL^−1^. Total overlapping circles were made in triplicates for each effector construct. Plants were incubated in a growth chamber for 5 days, when leaves with co-inoculated circles were taken for symptom analysis and photography. Three independent assays were performed.

### 2.10. Cloning, Bacterial Strains and Selection Conditions for EtHAn Conjugation and Infiltration

The *g3890* and *g5159* sequences were cloned into pDON221 and then recombined into pEDV6 vector [44]. In-frame fusions and the integrity of the effector sequences were confirmed by sequencing. *Escherichia coli* DH5α was used for propagating the pEDV6::*g3890* and pEDV6::*g5159* constructions, and then mobilized to *Pseudomonas fluorescens* EtHAn (*Pf* EtHAn) by standard triparental mating using *E*. *coli* HB101 (pRK2013) as helper strain. Transconjugated *Pf* EtHAn cells were selected in solid Luria-Bertani (LB) medium containing chloramphenicol (30 μg mL^−1^) and gentamicin (25 μg mL^−1^).

### 2.11. AvrB-Induced ETI Suppression Assay

Bacterial suspensions of *Pf* EtHAn expressing pVSP6::*AvrB* [45] and *Pf* EtHAn expressing the effector candidates individually (g3890 and g5159) were mixed (1:1 ratio) to a final of 2 × 10^7^ CFU mL^−1^ and co-infiltrated into four- to five-week-old *N. benthamiana* leaves using a needleless syringe. Co-infiltrations of *Pf* EtHAn (pVSP6::*AvrB*) and *Pf* EtHAn (pEDV6 empty) were used as negative controls in the same conditions. The level of suppression was estimated by an average percentage calculated on all infiltrated leaves in three independent experiments with eight biological replicates. 

## 3. Results

### 3.1. Sequence Features of the Selected Candidate Effectors

Following the methodology described (item 2.3), we selected 12 CE proteins for further investigation. They were classified into two classes according to their expression timeline as described previously [4]. The first class represents the CE most expressed during the early stages of infection and the second class those CEs potentially involved in more advanced stages of the disease (Table 1, Figure S3). This strategy enabled us to assess a range of candidates likely to be related to multiple processes during disease establishment and progression. Most CEs did not share conserved sequence features (domains) with other proteins, other than the canonical signal peptide (SP) for secretion (Table 1). The g3970 contains a glutamine–proline-rich region (PQ) followed by a cysteine-rich portion, and a complex array of variable repeats at the *C*-terminus (Figure S5). Three CEs (*g1052*, *g3890*, and *g5159*) were polymorphic among the isolates of *S. scitamineum* whose genomes were available (Table 1). The polymorphisms among the *S. scitamineum* sequenced isolates generated non-synonymous substitutions (Table 2; Figure S6).

### 3.2. CE Gene Expression during the Interaction

**Figure 1:**
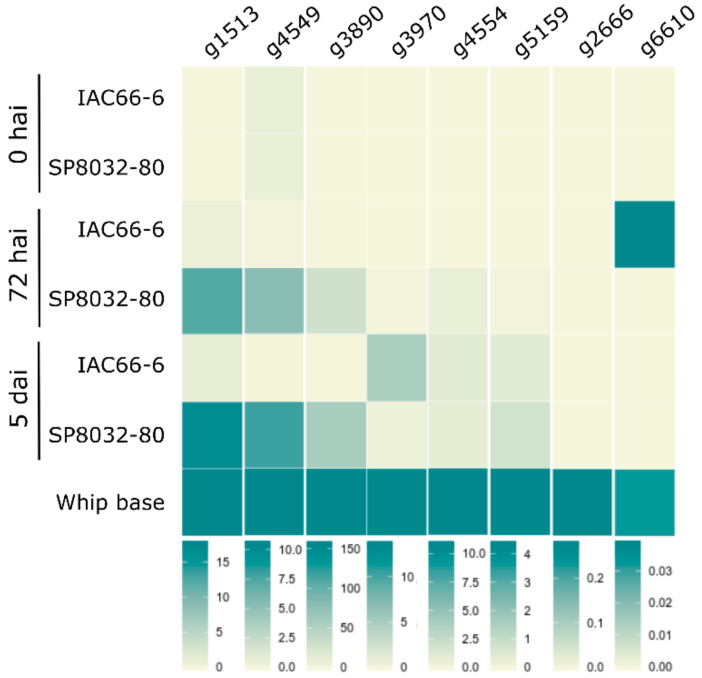
Candidate effectors (CE) transcriptional profiles of the fungus during disease progression (0 and 72 hai, 5 dai, and after whip emission) after infecting smut-susceptible (IAC66-6) and -resistant (SP80-3280) sugarcane plants. CEs were grouped based on similar transcriptional profiles. Vertical bars indicate scales of relative expression. Heatmaps were generated based on Figure S8 results.

We analyzed the expression of ten of the selected CE genes during disease progression. The pathogen colonized and grew in tissues of both the resistant (SP80-3280) and susceptible (IAC66-6) genotypes (Figure S7). Because we could not clone *g2* and *g4255* regardless of numerous attempts, and g1052 and g1084 showed unclear results for the expressed proteins fused with GFP, we dismiss these candidates from further discussions for the time being. According to the plant genotype, CE expression profiles varied over time (during disease progression) (Figure 1 and Figure S8). In general, the genes showed an earlier and stronger expression when the fungus interacted with resistant plants. None of the CE genes expressed when the fungus was grown in vitro, either as haploid yeast-like cells or as dikaryotic hyphae. They all expressed after fungal penetration (5 dai) and at the whip-base developed in susceptible plants (Figure 1 and Figure S8).

### 3.3. Sporisorium scitamineum Candidate Effectors Accumulate in a Variety of Plant Subcellular Compartments

**Figure 2:**
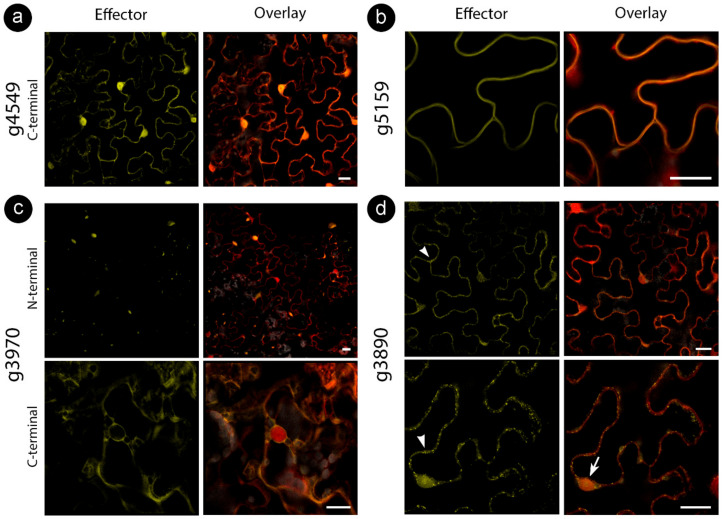
*Sporisorium scitamineum* candidate effector proteins accumulate in different subcellular compartments. All fusion proteins were co-infiltrated with free RFP to help detect cytoplasm and nucleus. *N*-terminal denotes the presence of an *N*-terminal YFP tag, and *C*-terminal denotes the *C*-terminal citrine fusion. Representation of (**a**) stable nucleocytoplasmic, (**b**) cell wall, (**c**) aggregations (upper panel) and cytoplasmic (lower panel), (**d**) cytoplasmic with vesicles (arrowhead in the upper panel), nucleolar (arrow in the lower panel) and mitochondrial (arrowhead in the lower panel) localizations. Bars: 20 µm.

We cloned the CE sequences without a signal peptide fused to either an *N*- or *C*-terminal fluorescent protein downstream of a 35S promoter in *A. tumefaciens* binary vectors (*N*-terminal YFP and *C*-terminal citrine). Proteins were transiently expressed in *N. benthamiana* epidermal cells and analyzed by confocal microscopy and immunoblots (Figure 2). 

**Figure 3:**
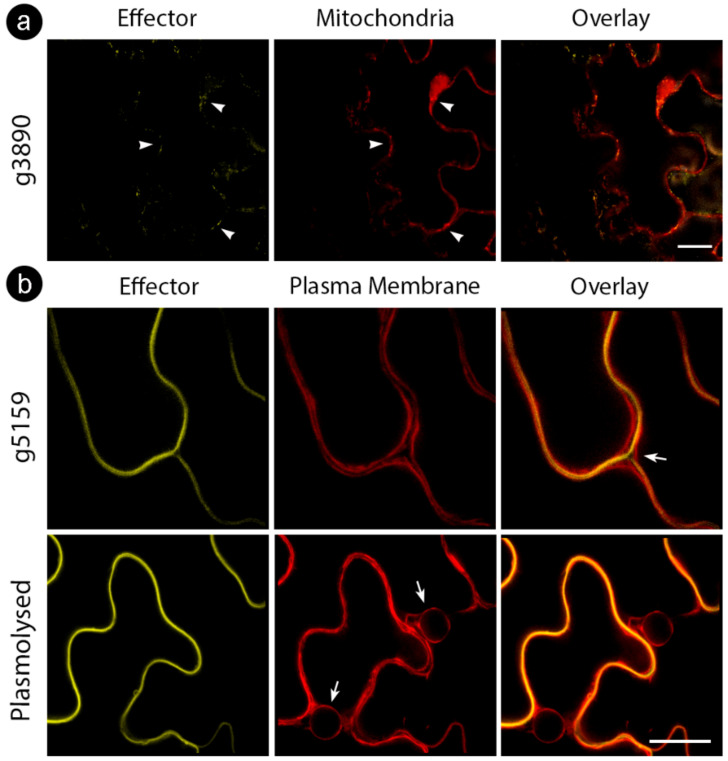
Cellular markers co-infiltrated with *S. scitamineum* candidate effector proteins. (**a**) g3890 co-infiltrated with mitochondrial marker, showing colocalization (white arrowheads) (**b**) g5159 co-infiltrated with plasma membrane marker before (upper row) and after (bottom row) plasmolysis with 0.5M NaCl. Note the localization of effector fusion protein between plasma membranes of adjacent cells (white arrow, upper row) and plasma membrane regression after plasmolysis (white arrows, bottom row). Bars: 20 µm.

While some proteins showed stable localization regardless of tag location, some varied the targeting patterns (Table 3). For those CEs delivered to specific compartments, such as the cell wall (for g5159) and mitochondria (for g3890), colocalization assays with cellular markers were performed to confirm specificity (Figure 3). We also explored localization with other cellular markers for CEs with inconclusive subcellular localizations (g3970 and g6610) fused with an *N*-terminal tag. However, colocalization did not reveal a particular compartment, suggesting protein aggregations (Figure 2c, upper panel). 

### 3.4. Sporisorium scitamineum Candidate Effectors Modulate Plant Immune Responses and Disease in Nicotiana benthamiana

**Figure 4:**
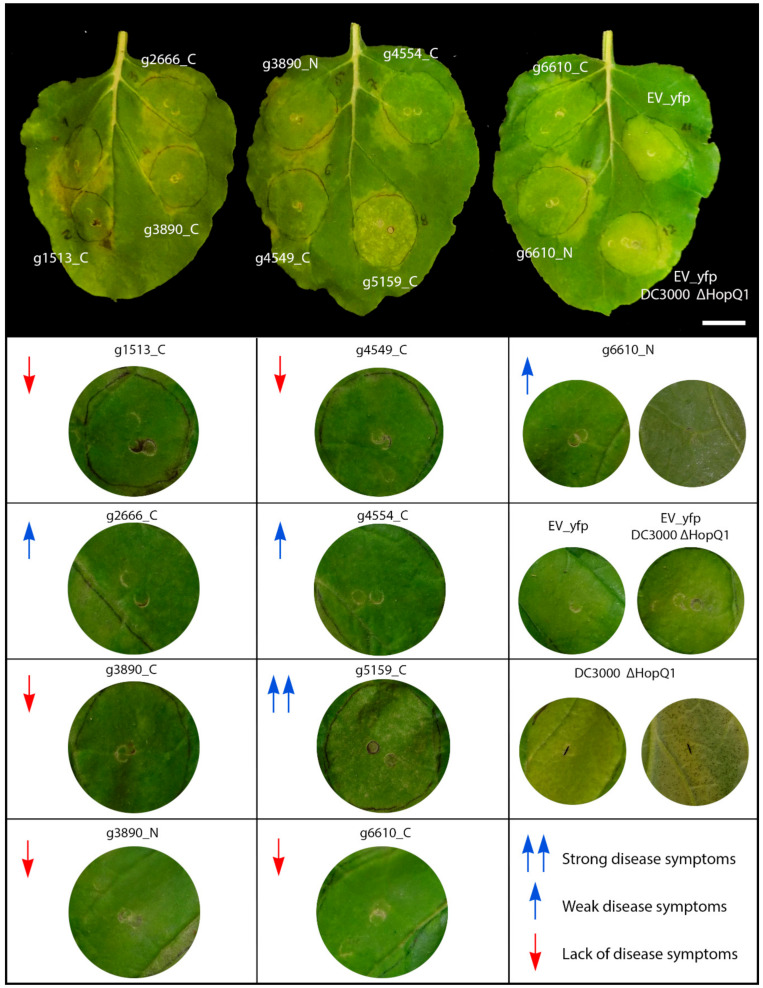
Virulence assay. The assay was conducted by the inoculation of *Pst* DC3000 ΔHopQ1-1 mutant over *S. scitamineum* candidate effector proteins expressed by agroinfiltration. Different levels of disease symptoms were observed. Red down arrows mean lack of disease symptoms, blue up arrows weak disease symptoms and double blue up arrows strong disease symptoms. Bar: 2.5 cm.

We tested the influence of *S. scitamineum* CE proteins in suppressing basal defense responses and disease symptoms using the model *N. benthamiana–P. syringae* pathosystem. The leaves were co-infiltrated with the deletion mutant strain of *P. syringae* pv. *tomato* ΔHopQ1-1 (*Pst*) with the *C*-terminal-targeted fusion proteins, in which the subcellular location was consistent, and both the *N*- and *C*-terminal versions of those whose localizations varied. This *Pst* strain causes disease in *N. benthamiana* when inoculated with a high concentration of cells (10^8^ CFU mL^−1^) [48]. The symptoms involved the abnormal yellowing of leaf tissue observed 5 days after *Agrobacterium* EV–YFP infiltration (negative control), the typical bacterial speck lesions produced by the *Pst* mutant, or both in the inoculation zone (Figure 4).

The set of CEs (g2, g1513, g3890, both *N*- and *C*-terminal versions; g4549 and the *C*-terminal tagged g6610) did not exhibit disease symptoms (Figure 4, red down arrows). Conversely, g5159 strongly increased *Pst* disease symptoms with the aggressive production of water-soaked lesions and chlorotic spots (Figure 4, double blue up arrow). The effectors g2666, g4554, and the *N*-termini tagged version of g6610 showed weaker disease symptoms (Figure 4, blue up arrows).

### 3.5. Interactors of S. scitamineum Effector Candidates

We sought to identify interacting partners through co-immunoprecipitation assay followed by liquid chromatography–tandem mass spectrometry (CoIP/LC-MS/MS) after transient expression in *N. benthamiana* leaves. We selected two of the CEs, g3890 and g5159 (*C*-terminal fusion versions), for further analysis since they showed the most contrasting results in previous assays and a better pattern of immunoprecipitation (Figure S10). The protein sequence resulted in 209, 162, and 240 proteins identified in the control, g5159, and g3890 samples, respectively. Manual inspection comparing control samples with each of the CEs allowed the identification of 26 proteins interacting with g5159 and 81 with g3890. All the interacting candidates showed a considerable number of unique peptides mapped in all triplicates. We then selected only distinct interacting partners for each CE showing at least three unique peptides and high scores (Score ≥ 5) for further discussion. The g5159 showed five specific interaction partners, while the g3890 was associated with 42 proteins distributed in 21 groups of plant proteins (Tables S5 and S6).

Sugarcane orthologs of the *Nicotiana* genes coding proteins found as interacting partners of both CEs were investigated to derive further evidence about their role in sugarcane responses to the pathogen (Tables S7 and S8). Among the interactors, only four orthogroups were predicted as having single orthologs in sugarcane for the interaction partners of g3890: (1) elongation factor 1-gamma 2-like isoform X2; (2) auxin transport protein BIG; (3) clathrin heavy chain 1-like; and (4) carbamoyl-phosphate synthase large chain. For g5159, only one orthogroup was identified with a single ortholog (succinate dehydrogenase, and protease 1). The remaining candidates had more than one ortholog. *Sorghum*, recurrently used as a genomic reference for sugarcane, was also found harboring single ortholog copies for some of the interaction partners (Tables S8 and S9).

**Figure 5:**
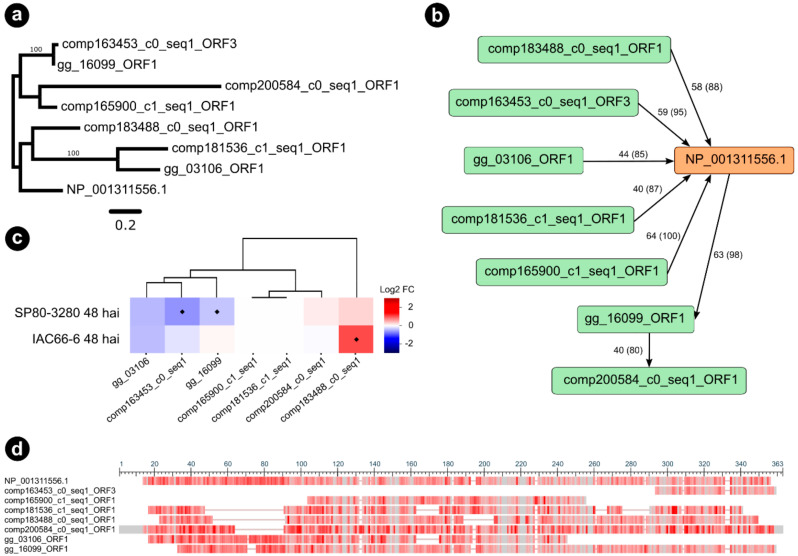
Sugarcane orthologs from orthogroup 5159_6 of *Nicotiana tabacum* (NP_001311556.1) endochitinase A precursor identified as interacting with g5159. (**a**) Maximum likelihood phylogenetic tree of endochitinase A gene family in sugarcane and *Nicotiana tabacum*. Nodal support values are given as posterior probabilities (%) above the branches if > 85%. (**b**) Graph showing the predicted orthogroup, with nodes depicted as green and orange rectangles representing sequences, and edges depicted as black arrows connecting nodes if they established a query-subject BLASTp relationship. Queries are at the source of arrows, whereas subjects are at the target of arrows. Edges labels represent BLASTp percentage of identity and query coverage. (**c**) Heatmaps showing expression profiles of sugarcane genes from orthogroup as values of a log2 fold change (inoculated/control) across two transcriptome experiments. Blue colors indicate downregulation, whereas red colors indicate upregulation. Diamonds represent statistical significance of expression at *p* < 0.05. (**d**) Multiple sequence alignment overview of the orthogroup obtained with the NCBI “Column Quality score—Protein” method showing conserved regions in grey bars, and alignment gaps as grey lines. Infrequent bases are highlighted as a red color gradient where darker red means more frequent and lighter red less frequent amino acids.

Among the selected interaction partners of the *S. scitamineum* candidate g5159, we found a plant endochitinase A, a promising candidate considering the identification score, which we further investigated. One orthogroup (5159_6), including seven orthologous sequences of the endochitinase A, was identified (Figure 5a,b).

According to our maximum likelihood phylogeny of the orthogroup 5159_6 (Figure 5a), three sugarcane sequences were closely related and grouped within the same clade as *Nicotiana* endochitinase A (P08252). All the three closely related sugarcane sequences were predicted as orthologs of endochitinase A during BLASTp searches using the sugarcane ORFs dataset as queries (Figure 5b). Interestingly, the genes encoding for comp163453_c0_seq1_ORF3 and gg_16099_ORF1, respectively showing 72% and 76% similarity (percentage of positive-scoring matches) to endochitinase A (NP_001311556.1), were downregulated 48 hai in the resistant genotype SP80-3280 (Figure 5c). Conversely, another orthologous gene encoding for comp183488_c0_seq1_ORF1 (79% similarity) was highly induced in the 48 hai experiment with the susceptible genotype IAC66-6. The multiple sequence alignment of orthogroup 5159_6 (Figure 5d) shows conserved regions, alongside the Pfam Glycoside hydrolase family 19 domain (PF00182), which supports both the analysis of orthology and the observed differential expressions of the gg_16099_ORF1 and comp183488_c0_seq1_ORF1 genes. However, the sequence of comp163453_c0_seq1_ORF3 represented only a fragment of the 62 amino acid residues of endochitinase A. We performed a broader ML phylogeny inference of endochitinase orthologs by including sequences from another sugarcane experiment (*N* = 10) [49] as well as from *A. thaliana* predictions (*N* = 24) [50]. PfamScan v39 [51] and SignalP v5 [52] softwares were used to research the structural features of endochitinase orthologs. Our consensus tree (Figure 5a,c) reinforced our observations for the three endochitinase orthologs found as differentially expressed (gg_16099_ORF1, comp163453_c0_seq1_ORF3, and comp183488_c0_seq1_ORF1). A single clade (branch support PP 88%) grouped the three mentioned orthologs together with the *Nicotiana* endochitinase A sequence (NP 001311556.1), the sequences of sugarcane endochitinase class I (ScChiI1, ScChiI2, ScChiI3), in addition to *A. thaliana* sequence of class I (AT3G12500.1). Nested within the mentioned clade, the sugarcane sequence of class II (ScChiII1) grouped together with the comp183488_c0_seq1, forming a single clade (branch support PP 100%).

Among the 42 interacting partners of the CE g3890, the most promising interacting candidates were orthologs from three subunits belonging to a type 2A protein phosphatase (PP2A) complex (Figure S11). Based on our ML phylogeny inference, including *Nicotiana* and *A. thaliana* sequences [53], two of the orthologs grouped with the regulatory subunit clades of B and B′, and one other ortholog grouped with a catalytic subunit A clade. Their expression profiles, based on previously obtained RNAseq data, showed all three orthologs as downregulated in the experiment with the smut-resistant sugarcane. Other potential interactors of g3890 were the T-complex protein 1 (beta, epsilon, theta, eta chains), ferritin and importin-5-like.

### 3.6. Bacterial Delivery of the Effector Candidate g5159 from S. scitamineum Suppresses AvrB-Induced ETI in Nicotiana benthamiana

**Figure 6:**
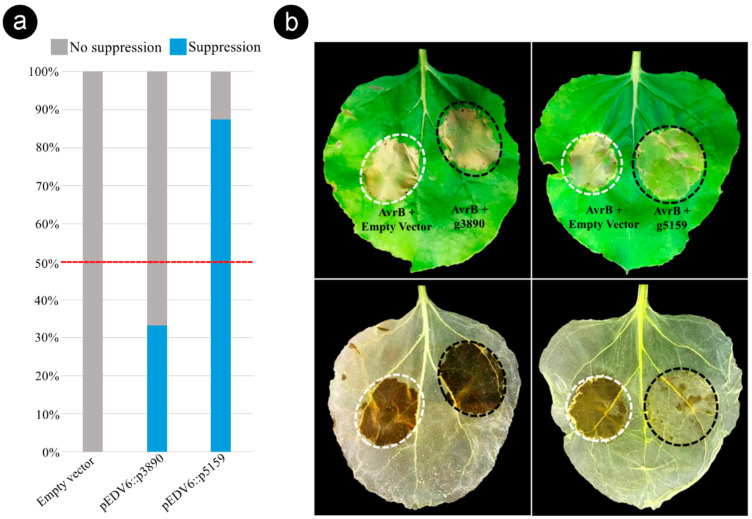
*Sporisorium scitamineum* effector candidate g5159 suppresses AvrB-induced effector-triggered immunity (ETI) in *Nicotiana benthamiana*. (**a**) Histogram displaying results of the ETI suppression assay performed with the pEDV6::*g3890* and pEDV6::*g5159* constructs. Level of suppression was estimated by the percentage frequency calculated on all co-infiltrated leaves in three independent experiments with eight biological repetitions (Table S9). Effector candidates that suppressed tissue collapse in more than 50% of co-infiltrated leaves were considered as ETI suppressors. (**b**) Phenotypic outcome from the ETI suppression assay. Bacterial suspensions of *Pseudomonas fluorescens* EtHAn expressing pVSP6::*AvrB* and *P. fluorescens* EtHAn expressing pEDV6 empty (AvrB + Empty Vector) were mixed (1:1 ratio) to a final of 2 × 10^7^ CFU mL^−1^ and used as controls (white dotted circle). Suspensions of *P. fluorescens* EtHAn expressing pVSP6::*AvrB* and *P. fluorescens* EtHAn expressing individually the effector candidates (AvrB + g3890) and (AvrB + g5159) were co-infiltrated under the same conditions (black dotted circle). Lower panel, same leaves decolorized by alcohol bleaching to visualize cell death. Photographs taken at 72 h after co-infiltrations.

To investigate whether the g3890 and g5159 tested in this study were capable of suppressing ETI responses, we established a specific ETI suppression assay by the co-delivery of AvrB and CEs via the Type Three Secretion System of the nonpathogenic *P. fluorescens* Effector-to-Host Analyzer (EtHAn) strain into *N. benthamiana* cells. We first analyzed the ability of *Pf* EtHAn to translocate AvrB into *N. benthamiana* cells. AvrB-dependent HR (ETI response) was visualized in the area infiltrated with *Pf* EtHAn expressing pVSP61::*AvrB*, whereas *Pf* EtHAn containing pEDV6 empty vector and *Pf* EtHAn non-transformed (negative controls) did not induce HR (Figure S12). We then evaluated whether the two CEs g3890 and g5159 could suppress the tissue collapse associated with AvrB-induced cell death (Figure 6). 

Based on the phenotypic contrast outcome observed 48–72 h after the co-infiltration of AvrB and individual CEs (1:1 ratio), we found that the effector candidate g5159 suppressed the tissue collapse induced by AvrB in the sites co-infiltrated in 87.5% of tested leaves (Figure 6 and Table S9). In contrast, the candidate g3890 presented a lower ETI suppression activity (33.3%) (Figure 6). The leaves were decolorized by alcohol bleaching to ensure accurate observation of the tissue collapse (Figure 6b). These results show that g5159 can suppress AvrB-induced ETI responses in *N. benthamiana*, suggesting that *S. scitamineum* produces effector proteins to inactivate HR cell death as a strategy for subverting the sugarcane immune system.

## 4. Discussion

### 4.1. General Aspects of the Candidate Effectors

Effector biology has become a powerful tool in understanding pathosystems and direct plant breeding programs [10,35,54]. There are only a few studies considering effectors encoded by *S. scitamineum* and their expression profiles during disease progression [4,5,55,56,57,58,59]. This work provides further insights into the sugarcane–smut pathosystem by leaping into effector biology.

With the advent of “omics” and the use of bioinformatics techniques, accessing the fungal effector repertoire became a more feasible task. For predicting effector candidates, most methods rely on an *N*-terminal signal peptide in cysteine-rich small proteins. Despite being helpful to track apoplastic effectors, the cytoplasmic effectors can be larger proteins with lower cysteine content. This class of proteins for filamentous fungi is still poorly understood [54]. Due to the frequent lack of known sequence signatures and the high level of variability even in closely related groups, defining the effector repertoires tends to be less rigid in fungal effector biology. More recently, the structure-based analysis of effectors revealed protein structural conservation at the three-dimensional level [54].

Smut fungi use a cocktail of effectors in waves of expression over time to maintain their biotrophic growth throughout their life cycle [60,61,62,63,64]. Based on previous work, we selected candidate effectors that varied their expression when infecting susceptible plants at 5 dai and at 200 dai, after whips emerged [4]. Among the selected candidates, we chose the ones with the highest number of mapped reads (preferentially expressed) and stage-dependent expression varying among plant genotypes [60]. As determined for other characterized effectors [65], none were expressed in axenic culture, suggesting that they all play a potential role in plant–pathogen interactions. Moreover, most of them were unique to the *S. scitamineum* genome, encoding proteins relevant for the sugarcane–smut interaction. Although we suggest that the selected genes encode candidate effectors, we acknowledge that further functional characterization must complement the studies presented here. Mutant generation for specific candidates is one of the most relevant strategies, in addition to the subcellular location of the candidates in sugarcane cells delivered directly by the fungus. However, this first approach shortened the list of candidates to be tested in experiments using sugarcane plants.

For some loci associated with the plant–pathogen arms-race, polymorphisms may influence the outcome of interactions [66], considering not only inter- but also intraspecies population studies [67,68]. Investigating the sequence variation among the CEs of the four sequenced genomes [4,59,69], Benevenuto et al. [27] unveiled polymorphisms in some of the candidates. For instance, g1052 presented two single nucleotide polymorphisms at positions 25 and 472, introducing non-synonymous substitutions. We extended this analysis for all the CEs and detected other polymorphic effectors, such as g3890 and g5159. Curiously, the Brazilian and South African isolates showed similar modifications in g1052, while Australian and Chinese strains shared the same sequence features. Benevenuto et al. [47] revealed two haplotypes, which seem to maintain the same pattern in a population of 50 Brazilian isolates we have been investigating (unpublished data). The effector candidates in which we detected a unique localization pattern and expression in resistant plants (g3890 and g5159) showed polymorphic loci producing nonconservative amino acid substitutions. Polymorphic effectors are potentially related to the presence of races [20]. Although the presence of races in *S. scitamineum* is still controversial, some authors have defined races in specific populations infecting sugarcane [1,70]. It remains undetermined whether these two haplotypes have differences impacting the outcome of the interaction.

### 4.2. The Expression of S. scitamineum Effector Genes Is Dependent on the Host Genotype

The approach we used was complementary to the one used by Barnabas et al. [58]. These authors determined the transcriptional profile of six smut core effectors (Pep1, Cmu1, Tin2, Srt1, Stp1, and Pit1) functionally characterized for the *U. maydis*–maize pathosystem. They all had a similar expression pattern infecting the host tissue and did not express in axenic culture. In our study, however, the overall expression data suggested that we selected candidates involved in different mechanisms used by *S. scitamineum* to colonize sugarcane tissues. Biotrophs, in general, use various strategies [71,72], secreting effectors throughout the life cycle even before contact with the plant surface [73,74,75,76]. Some others are induced upon infection, requiring the activity of specific transcriptional factors [77,78,79]. These effectors are involved in avoiding basal plant defenses and, at the same time, in inducing extensive modifications in the host cell metabolism [80]. According to the candidate’s expression pattern, we identified three categories: (1) late expression only in the susceptible genotype (*g2666*, *g3970*, and *g6610*); (2) early and high expression in the resistant genotype (*g1513*, *g3890*, and *g4549*); and (3) expression in both resistant and susceptible genotypes (*g1052*, *g1084*, *g4554*, and *g5159*).

Previously, we determined that *S.scitamineum* delayed germination and appressorium formation in resistant plants, which were associated with the induction of the host oxidative burst and the fungal antioxidant system [25,26]. These responses differed in infected susceptible plants and fungal axenic growth. The *S. scitamineum* lifestyle displays an initial infection phase that encompasses germination, appressorium formation, penetration, and meristem colonization. In this case, colonization does not always result in disease, and the time taken for this initial phase varies according to the plant genotype [24,25]. 

In our data, the host genotypes influenced the candidates’ expression profiles. For instance, the induction of *g3890* after 72 hai only in resistant plants agreed with the increased hydrogen peroxide content (70%) in this same resistant genotype infected with *S. scitamineum* [25]. Although further experiments are necessary, the association of *g3980* with ROS induction stands out as a valid hypothesis. We detected a similar expression pattern for *g1513* and *g4549*. Other candidates (*g1052*, *g1084*, *g4554*, and *g5159*) were expressed in both resistant and susceptible plants.

The expression profiles of *g2666* and *g6610* in susceptible plants exclusively at a late stage in association with whip emission, and the expression pattern for *g3970* after five dai only in susceptible plants, were notable. The role of these CEs expressed later may be related to fungal sporogenesis. The *U. maydis* ortholog of *g3970*, Rsp3, had its function described at the time of our work [46]. However, the protein encoded by *g3970* is a larger version. The gene also has length polymorphisms between different alleles, as described for the *U. maydis* gene. The polymorphic region, expanding from position 181 to 240, includes aromatic residues known to stack and be highly hydrophobic. The Rsp3 protein is secreted and attached to the hyphal surface to block the maize mannose-binding proteins’ antifungal activity and the recognition by R proteins [46]. The expression of *Rsp3* peaked two dai in *U. maydis*, whereas for *S. scitamineum*, expression was detected five days after inoculation, reaching its maximum after whip emission. Consistent with Rsp3, the effect of g3970 potentially also protects the fungus when infecting susceptible plants. However, its specific role in the sugarcane–smut pathosystem remains elusive.

### 4.3. S. scitamineum Effector Proteins Target Multiple Cell Compartments

The set of nine effector candidates studied for subcellular localization with *N*- and *C*- terminal fusions allowed separation into two groups: (1) stable localization of the proteins using either *C*- or *N*-terminal tags (g2, g2666, g4549, g4554, and g5159), and (2) proteins with differential localization using different tags (g1513, g3890, g3970, and g6610). Additionally, we grouped the candidates by their localization pattern: (A) nucleocytoplasmic proteins (g2, g1513, g2666, g4549, g4554), (B) proteins with compartment-specific localization (g3890 and g5159), and (C) proteins that aggregate or remain soluble in the cytoplasm (g3970 and g6610).

The targeting of various cellular compartments and many pathways is not unusual for filamentous pathogens [35]. Various effectors are nucleocytoplasmic, but can also act in multiple compartments [81,82]. The g1513 showed nucleocytoplasmic localization with an *N*-terminal tag and with a *C*-terminal fusion accumulated only inside the nucleus. The uneven localization pattern may indicate a functional domain responsible for nuclear uptake in the *N*-terminus. Nuclear effectors can coordinate activators or repressors of signaling pathways [17], while cytoplasmic effectors may function as direct or indirect interactors with resistance proteins [83].

As an ortholog, the *S. scitamineum* g3970 shared amino acid features with Rsp3. Along with g6610, it showed a similar localization pattern to that described for Rsp3, forming aggregates when the tag was *N*-terminal, and being cytoplasmic when the *N*-terminus was free. Rsp3 presents dual functions; the *N*-terminus is responsible for fungal cell wall interaction, and the *C*-terminus for interacting with the plant-derived protein AFP1. When the mature protein’s *N*-terminus is not available for interaction, the proteins may be aggregating because the fungal cell wall’s partners were not present. Otherwise, when the *C*-terminus was accessible, the protein was solubilized in the cytoplasm. Like Rsp3, the encoded protein of g6610 also contained two predicted disordered domains [84] between amino acids 45–79 and 92–134. Both g3970 and g6610 had similar expression patterns, induced only in susceptible plants. It is noteworthy that g3970 is also the most expressed gene of the *S. scitamineum* secretome in the susceptible genotype [4]. These observations show promising functions for these two candidates.

The protein encoded by g3890 tagged at the *N*-terminus displayed nucleocytoplasmic localization. However, when the tag was *C*-terminal, the proteins accumulated in the nucleolus and mitochondria. Other pathosystems revealed similar results for some effectors [85,86]. Chaudhari et al. [87] showed that even when GFP influences the subcellular location of tagged effectors due to its size and properties, nucleolar-localized GFP-tagged effectors are genuine since GFP does not transit to the nucleolus. The effector 30D08 from the sugar beet cyst nematode targeted host nucleoli, directly interacting with SMU2 (homolog of suppressor of mec-8 and unc-52 2), an auxiliary spliceosome protein. It interferes with the host splicing patterns altering mRNA processing and the expression of genes to influence plant susceptibility [86]. HaRxL44, secreted by *Hyaloperonospora arabidopsidis*, also targets the nucleolus, driving the host protein MED19a to proteasome degradation. MED19a appears to change salicylic acid-responsive defense transcription to jasmonic acid- and ethylene-responsive transcription, enhancing host susceptibility [87].

The g5159 explicitly localized to the plant cell wall. Its expression was induced 72 hai in the resistant variety, whereas the susceptible genotype delayed induction until 5 dai. Few functionally described effectors target the plant cell wall. Liu et al. [88] verified that HaEXPB2, an expansin-like protein secreted by the cyst nematode *Heterodera avenae*, localized to the plant cell wall when transiently expressed in *N. benthamiana* cells. The g5159 has no expansin-like domain, and the only sequence feature found was a small disordered domain at the *N*-terminus. It is noteworthy that the accumulation of this effector in the leaves of *N. benthamiana* was coincident with some loosening in the plant tissues (unpublished work) and prominent symptoms in the virulence assay performed after disease induction with the *P. syringae* mutant ΔHopQ1-1. Together, these findings suggest an influence in both the plant immune system and in pathogen colonization.

Petre et al. [35] and Petre et al. [89] described the effector CTP1 from *Melampsora larici-populina* to accumulate in both mitochondria and chloroplasts. The g3890 protein did not have a canonical transit peptide sequence. However, these motifs were more related to conserved physical properties than sequence features to prevent plant perception [90]. The *g3890* is one of the more highly expressed genes during the infection of resistant plants. Mitochondria are associated with ROS homeostasis and, especially for biotrophic pathogens, they are likely to manipulate ROS production and scavenge pathways to maintain balance for their own benefit [91,92]. Since resistant sugarcane plants respond to the infection via a robust oxidative burst 72 hai, the candidate g3890 targeting the mitochondria is likely to be related to this defense response [25].

### 4.4. g5159 Interactors and Suppression of ETI

The protein identified as the most promising interactor of g5159 in leaves of *N. benthamiana* was an endochitinase (P08252), which showed the highest score based on the number of unique peptides matching the protein sequence. The sequence is 72% similar to *Arabidopsis* At3g12500, having two transient peptide domains, the *N*-terminal sequence encoding the signal peptide for secretion, and the *C*-terminus encoding a vacuolar targeting signal, targeting the protein to the vacuole [93]. Other relevant orthologs were the SchiII1 of sugarcane [49] and the *S. bicolor* chitinase 2 (A0A1Z5R3E0_SORBI). This type of chitinase (EC 3.2.1.14) belongs to class 1 and uses a basic mechanism of substrate-assisted catalysis [50].

In our sugarcane sequence ORFs database [43], we identified the transcript comp183488 as an ortholog of the *N. benthamiana* gene, along with other plant orthologs. Chitinases classified as PR proteins play a significant role in defense against fungal pathogens, and belong to the glycoside hydrolases family 19 (http://www.cazy.org/GH19.html) [94]. Different plant chitinases have their expressions affected by various biotic and abiotic agents [94,95,96]. As described by Su et al. [49], the expressions of sugarcane chitinases, and especially SchII1, in plants infected with *S. scitamineum* showed variant profiles in resistant and susceptible genotypes. Resistant plants had an earlier expression (24–48 hai), whereas susceptible plants had a later expression (48–120 hai). Our RNAseq data showed similar results 48 hai, when susceptible plants strongly induced the endochitinase P08252 ortholog’s expression (comp183488 ortholog had a log2 fold change expression value greater than 1). On the other hand, two endochitinase orthologs were downregulated in the resistant genotype experiment. Further, although the fungus induced the effector g5159 in both resistant and susceptible sugarcane genotypes, an earlier expression was observed only in the resistant plants (48 hai). The g5159 localized attached to the cell wall in *N. benthamiana* cells, whereas the endochitinase is a vacuolar enzyme that is also secreted. To date, the only effectors identified targeting plant cell walls were associated with cereal cyst nematode infections [88]. As described for the HaEXPB2 effector protein of the cereal nematode, the candidate effector g5159 suppressed ETI, in addition to noticeably exacerbating disease symptoms in the virulence assay. Thus, the CE g5159 is our most encouraging candidate for further studies. 

We identified other candidate interactors for g5159, including four subunits of the 26S proteasome regulatory subunit. Recent studies have suggested that proteasome and autophagy pathways are central hubs for microbial effectors [97]. In addition, the monothiol glutaredoxin S17, the chaperone dnaJ protein homolog (HSP40), and a peroxiredoxin were all interacting candidates with a considerable number of unique peptides mapped in all replicates.

### 4.5. g3890 Targets Multicellular Compartments Potentially Having Multiple Partners

Unlike g5159, we were not able to narrow down the interactome of g3890 to a few candidate interactors in our experiment (Table S5). The identification of 42 potential interactors of different functional categories may be related to its multiple subcellular locations. Some of the potential interactors were orthologs of the same protein, and others were different subunits of the same protein complex.

For instance, we identified three subunits of a type 2A protein phosphatase (PP2A) complex, two isoforms of subunit B (B and B′), and one isoform subunit A. The PP2A belongs to a metal-independent protein phosphatase family involved in signaling many eukaryotic cell events, including pathogen defense [98]. The various isoforms of each subunit of the heterotrimer complex PP2A are responsible for the multiple localizations and functions of PP2A [99]. The subunit B is responsible for specificity, while subunits A and C are catalytic. We identified two different isoforms of subunit B, which would be the target of specific events involving PP2A. The sugarcane orthologous genes we detected in our database had an average of 78% similarity, and were supported by a consensus tree.

The g3890 expression induction occurred only in resistant plants 72 hai. In our RNAseq data 48 hpi, we detected transcripts encoding orthologs of the PP2A subunits downregulated in sugarcane plants resistant to smut. As mentioned before, Peters et al. [25] detected a 23% increase in hydrogen peroxide 48 hai, and a 70% increment 72 hai only in resistant sugarcane plants. Protein phosphatases are redox-regulated and potentially related to responses involving ROS as signaling molecules [99]. In experiments involving *Arabidopsis* and *Botrytis cinerea,* the inactivation of a PP2A impaired the proliferation of the fungus, and the MAPK activity was strongly induced [100]. Similar results were presented by Zhu et al. [101] for the pathosystem *Triticum aestivum* and *Rhizoctonia cerealis*. However, these are necrotrophic pathogens, and although we observed reduction in *S. scitamineum* proliferation in the resistant plants, sugarcane tissues are still colonized.

Regarding the induction of HR, He et al. [102], using *N. benthamiana* silenced for PP2A, and co-expressing an effector Avr9 from *Cladosporium fulvum* along with the known resistance gene of tomato *Cf9*, observed a rapid and robust HR followed by massive collapse of the infiltrated tissues. Using a wild-type *N. benthamiana* and the EtHAn system, g3890 did not suppress HR in our experiments. We did not test for PTI suppression. However, Zhu et al. [101] determined the role of PP2A in the suppression of PTI-related responses against *R. cerealis* [101]. 

Another set of proteins identified in our CoIP experiments included four subunits of the chaperonin-containing T-complex protein 1 (beta, epsilon, theta, eta). Interestingly, Ahn et al. [103] identified a regulatory subunit of PP2A (tap46) interacting with the T-complex in experiments using Arabidopsis. Similarly, He et al. [102] also reported the physical interaction of the PP2Aa regulatory subunit with the Pti1 kinase through Y2H assay. Here, we found an adenosine kinase 2, also described to be targeted and inactivated by the geminiviruses pathogenicity proteins AL2 and L2 [104]. The maize-smut fungus *U. maydis* is known to target hubs in the host immune system where they perform various tasks, which span from metabolism manipulations to developmental changes. These observations reinforce the putative roles g3890 might be playing in the phosphorylation status during disease establishment in sugarcane, especially in resistance genotypes.

Considering the two discussed g3890 interactions of PP2A, T-complex, and adenosine kinase 2, alongside the other 24 identified interactions, we believe to have derived consistent results for the cytoplasmic localization of this CE. However, interactors in the nucleolus and mitochondria, where we also localized g3890, were scarce. We detected five importin-5-like proteins which are localized in the nucleus and in the mitochondria, and a lipoamide acyltransferase component of the branched-chain alpha-keto acid dehydrogenase complex. Although all the interactions detected need further investigation, they are promising candidates and open up numerous opportunities to decipher the roles *S. scitamineum* effectors play during sugarcane interaction.

## 5. Conclusions

To the best of our knowledge, this study offered the first comprehensive analysis of *S. scitamineum* candidate effectors and their expression profiles in infected sugarcane plants of contrasting genotypes. We provide a set of 10 candidate effectors and determine the cell compartments where they may act in targeting plant proteins. The most promising candidates, g3890 and g5159, targeted different compartments and timeframes, resulting in outcomes that may contribute to disease resistance or susceptibility. For instance, the g5159 suppressed ETI in our conditions. Although we need further experiments, the scores used to select potential interactors suggested that the g3890 targets the signaling pathway involving PP2A, and g5159 potentially affects defense mechanisms via interaction with an endochitinase. 

Most importantly, we detected the sugarcane orthologs that may be the plant targets of these effectors. Our results were gained using model plants and expression data of sugarcane plants infected with *S. scitamineum*, providing indirect evidence of the functional role of effectors in the sugarcane–smut pathosystem. The technologies available for direct mutagenesis and the expression of effectors in transgenic sugarcane plants are of interest in order to detail the exact role of each of the candidates identified here. However, performing well-established experiments for model plants in sugarcane is time-consuming, and has a low success rate. Having a shorter list of candidates will improve our chances of a proof-of-concept for the direct functional characterization of *S. scitamineum* effectors in sugarcane, including the use of Co-IP (Protein complex immunoprecipitation) directly in sugarcane tissues. We are looking forward to the next steps in this endeavor of effector biology.

## Figures and Tables

**Table 1:** Candidate effector (CE) genes chosen based on their expression profile in two time points (see methods 2.3). Gene names in bold represent those CEs with no orthologs in other smut genomes.

Gene ID	CDS Size (nt)	Protein Size (aa)	Molar Weight (kD)	Effector Reference	Selection Features **	Genomic Island **	NLS	Cysteine %	Repeats	SNP	Disorder Domain	In Silico Location
g2	711	236	26,290	-	PE 5 dai; DE 200 dai; UF	x	x	2.50	x	x	✓	Apoplastic
g1052	423	140	15,539	[27]	Single.; EE; UF	x	x	2.86	x	✓	x	Non-apoplastic
g1084	930	309	34,473	-	Single., EE, UF	✓	x	0.00	✓ ^2^	x	✓	Non-apoplastic
g1513	648	215	23,140	[27]	PE 5 dai; DE 5 dai; DE 200 dai; UF	x	x	1.40	x	x	✓	Apoplastic
g2666	429	142	16,443	-	Single.; EE; UF	✓	x	0.00	x	x	x	Non-apoplastic
g3890	408	135	14,860	[27]	PE 5 dai; EE; UF	x	x	3.70	x	✓	x	Non-apoplastic
g3970	2238	745	79,540	[46]	PE 5 dai; DE 5 dai; DE 200 dai; UF	x	x	1.30	✓	✓	✓	Non-apoplastic
g4255	2301	766	86,484	-	Single.; EE; UF	✓	Monopartite Score 9 ^1^	0.78	x	x	✓	Non-apoplastic
g4549	345	114	12,406	[27]	Single.; EE; UF	✓	x	1.75	x	x	x	Non-apoplastic
g4554	396	131	14,068	[27]	Single.; EE; UF	✓	x	1.53	x	x	x	Non-apoplastic
g5159	1293	430	48,247	-	Single.; EE; UF	✓	x	0.23	✓ ^3^	✓	✓	Non-apoplastic
g6610	624	207	21,596	-	Single.; EE; UF	x	x	0.48	x	x	✓	Non-apoplastic

All genes have signal peptide and are induced during host interaction. bp: base pairs; aa: amino acids; dai: days after inoculation; DE: differentially expressed; PE: preferentially expressed; Single: *S. scitamineum* singleton; EE: exclusively expressed in planta at 200 dai; UF: unknown function. ** According to Taniguti et al. [4]. ^1^ monopartite NLS sequence. ^2^ sequence repeats “LGHRQpASAPAINAhLPVLTTD” and “PFADDAlleRLVLEFtKSRAQL” two times repeated each. ^3^ sequence repeats “PRMPlFQMERPDVN”, “ILPikPSDEQTQMLV” and “GHGPSTTEVVIHPLTRKERF” two times repeated each.

**Table 2:** Polymorphic loci positions in candidate effectors (CE) proteins.

Candidate Effector	PL1	PL2
g1052 ^1^	A9T	R158G
g3890	H35R	L112Q
g5159	Y230W	-

^1^ Described by Benevenuto et al. [47]. PL: polymorphic loci.

**Table 3:** Summary of the CE fusions presenting subcellular localization in *N. benthamiana* leaves.

Protein ID	Subcellular Location	
	*N*-tag	*C*-tag
g1513	Cytoplasmic	Nucleocytoplasmic
g2666	Nucleocytoplasmic	Nucleocytoplasmic
g3890	Nucleocytoplasmic	Nucleocytoplasmic ^1^, mitochondria
g3970	Aggregates	Cytoplasm
g4549	Nucleocytoplasmic	Nucleocytoplasmic
g4554	Nucleocytoplasmic	Nucleocytoplasmic
g5159	Cell wall	Cell wall
g6610	Aggregates	Cytoplasm

^1^ Nucleolar enrichment; *N*-tag and *C*-tag denote the site of the fluorescent tag.

## Supplementary Materials

The following are available online at https://www.mdpi.com/2309-608X/6/4/339/s1, Figure S1. Overview of the greenhouse experiment with inoculated sugarcane varieties. Figure S2. Sampling after whip emission. Figure S3. Venn diagram used for candidate effector selection. Figure S4. Dissociation curve generated by RT-qPCR reactions for endogenous control and target genes. Figure S5. Protein architecture of g3970. Figure S6. Sequence alignment of candidate effector orthologs from four different strains. Figure S7. *S. scitamineum in planta* quantification by qPCR. Figure S8. *In planta* expression profile of genes encoding *S. scitamineum* effector candidates by RT-qPCR analysis. Figure S9. Immunoblots prepared with total protein extracts. Figure S10. Immunoblot prepared with effector fusion proteins after immunoprecipitation with anti-GFP beads. Figure S11. Maximum likelihood phylogenetic tree showing the relationships among PP2A orthologs predicted within sugarcane, *Nicotiana*, and *Arabidopsis thaliana* genomes. Figure S12. AvrB activates effector-triggered immunity (ETI) in *N. benthamiana*. Table S1. Nucleotide sequences of the predicted CDSs (Coding DNA Sequences) from selected candidate effectors used in this study for functional investigation. Table S2. Primers designed for *S. scitamineum* candidate effector gene expression analysis by RT-qPCR. Table S3. Primers for effector cloning into Gateway system. Table S4. List of vectors used in this work. Table S5. List of unique interacting partners of the candidate effector g3890 obtained from CoIP/LC-MS/MS. Table S6. List of unique interacting partners of the candidate effector g5159 obtained from CoIP/LC-MS/MS. Table S7. The sugarcane orthologs of the *Nicotiana* genes coding for the proteins found to be interacting partners of the candidate effector g3890. Table S8. The sugarcane orthologs of the *Nicotiana* genes coding for the proteins found to be interacting partners of the candidate effector g5159. Table S9. *S. scitamineum* candidate effectors tested for ETI suppressing activity in *N. benthamiana*.
