# Supplementary material for: Leaping into the Unknown World of Sporisorium scitamineum Candidate Effectors
Source: J Fungi (Basel). 2020 Dec 4;6(4):339. doi: 10.3390/jof6040339 (PMC7762069; doi:10.3390/jof6040339)
Supplement: Supplementary file 1 [file jof-06-00339-s001.zip › Supplementary File 2.docx]

SUPPLEMENTARY FILE 2

Article

Leaping into the unknown world of *Sporisorium scitamineum* candidate effectors

Natália Sousa Teixeira-Silva ^1,2^, Patrícia Dayane Carvalho Schaker ^1^, Hugo Vianna Silva Rody ^1^, Thiago Maia ^1^, Christopher M. Garner ^2,3^, Walter Gassmann ^2,3,^* and Claudia Barros Monteiro-Vitorello ^1,^*

^1^ Universidade de São Paulo, Escola Superior de Agricultura “Luiz de Queiroz”, Departamento de Genética, Av. Pádua Dias, 11, CP 9, CEP 13418-900, Piracicaba, SP, Brasil; nataliasteixeirasilva@gmail.com, patriciaschaker@utfpr.edu.br hugorody@gmail.com, thiago.maia@usp.br, cbmontei@usp.br

^2^ Christopher S. Bond Life Sciences Center and Interdisciplinary Plant Group, University of Missouri, Columbia, MO 65211, USA; nataliasteixeirasilva@gmail.com, GassmannW@missouri.edu

^3^ Division of Plant Sciences, College of Agriculture, Food and Natural Resources, University of Missouri, Columbia, MO 65211, USA; GassmannW@missouri.edu, cmgarner07@gmail.com

* Correspondence: CBMV: cbmontei@usp.br; WG: GassmannW@missouri.edu


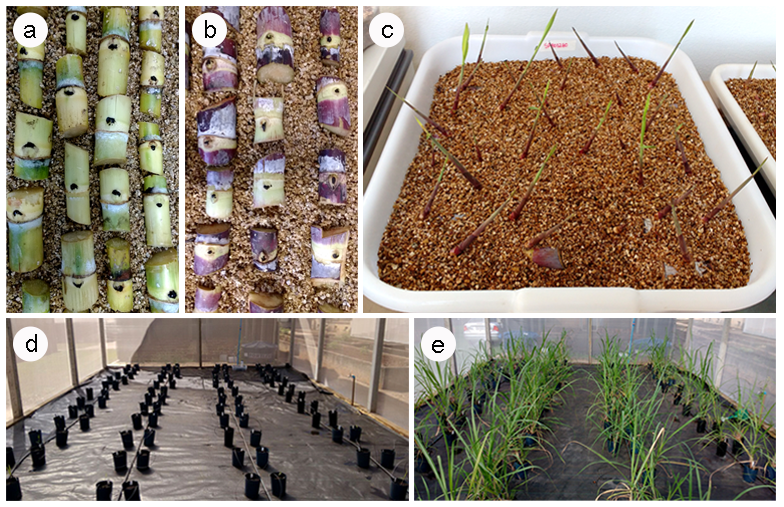


**Figure S1. Overview of the greenhouse experiment with inoculated sugarcane varieties.** Teliospore paste inoculation over IAC66-6 **(a)** and SP80-3280 **(b)** single buds. **(c)** Single buds two weeks after inoculation prior to transplantation. Conduction of experiment under greenhouse conditions at 20 days after inoculation (dai) **(d)** and 90 dai **(e)**.


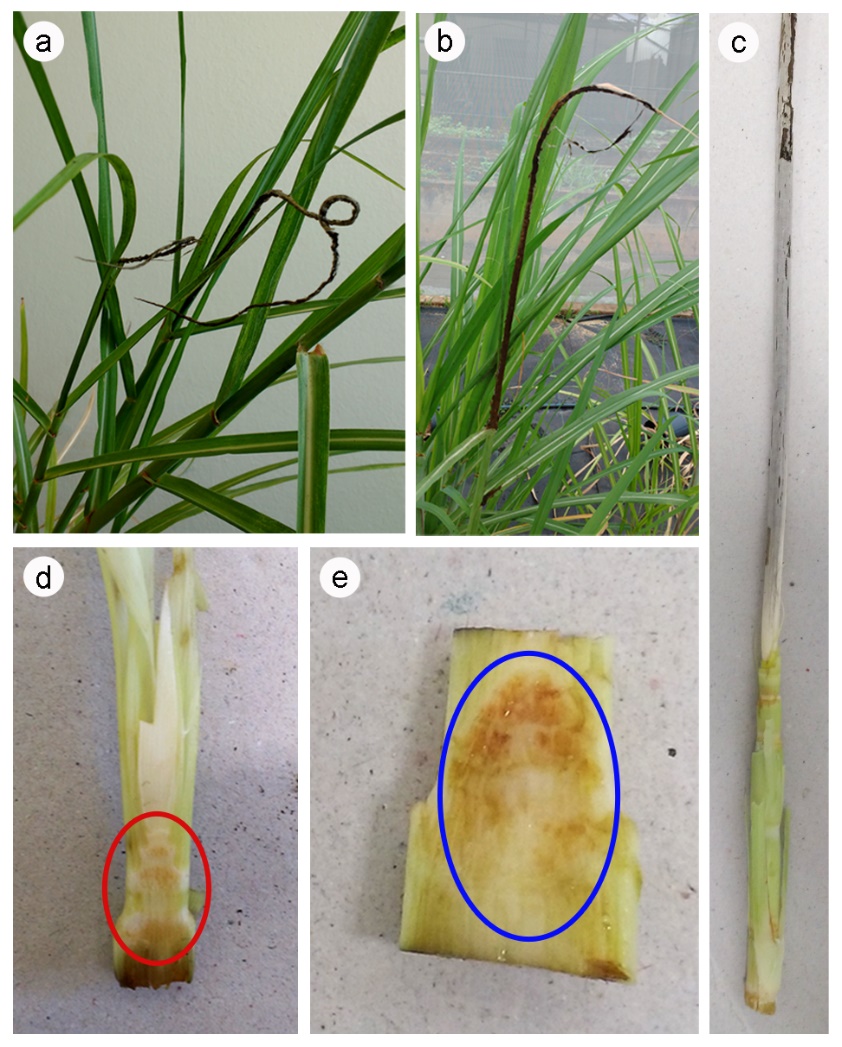


**Figure S2.** **Sampling after whip emission.** Sugarcane whips from IAC66-6 90 days after inoculation (dai). **(a)** and 140 dai **(b)**. Excised whip structure from IAC66-6 **(c)**. Sampled sugarcane meristem at the whip base of IAC66-6 (red circle) **(d)** and in infected sugarcane meristem of SP80-3280 without a whip (blue circle) **(e)**.


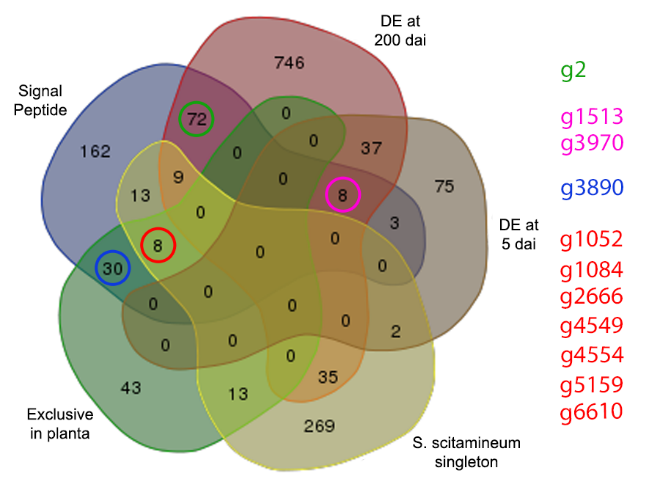


**Figure S3.** **Venn diagram used for candidate effector selection.** Based on the *S. scitamineum* annotated genome and the dual transcriptomic analysis [4, 5], the genes encoding proteins of the predicted secretome (Signal Peptide); genes differentially expressed during the interaction at five dai (DE at 5 dai) or 200 dai (DE at 200 dai); genes unique to the *S. scitamineum* genome (*S. scitamineum* singletons) and genes expressed only in planta (Exclusive in planta) were selected. Colors define the selected genes. DE (differentially expressed); dai (days after inoculation).


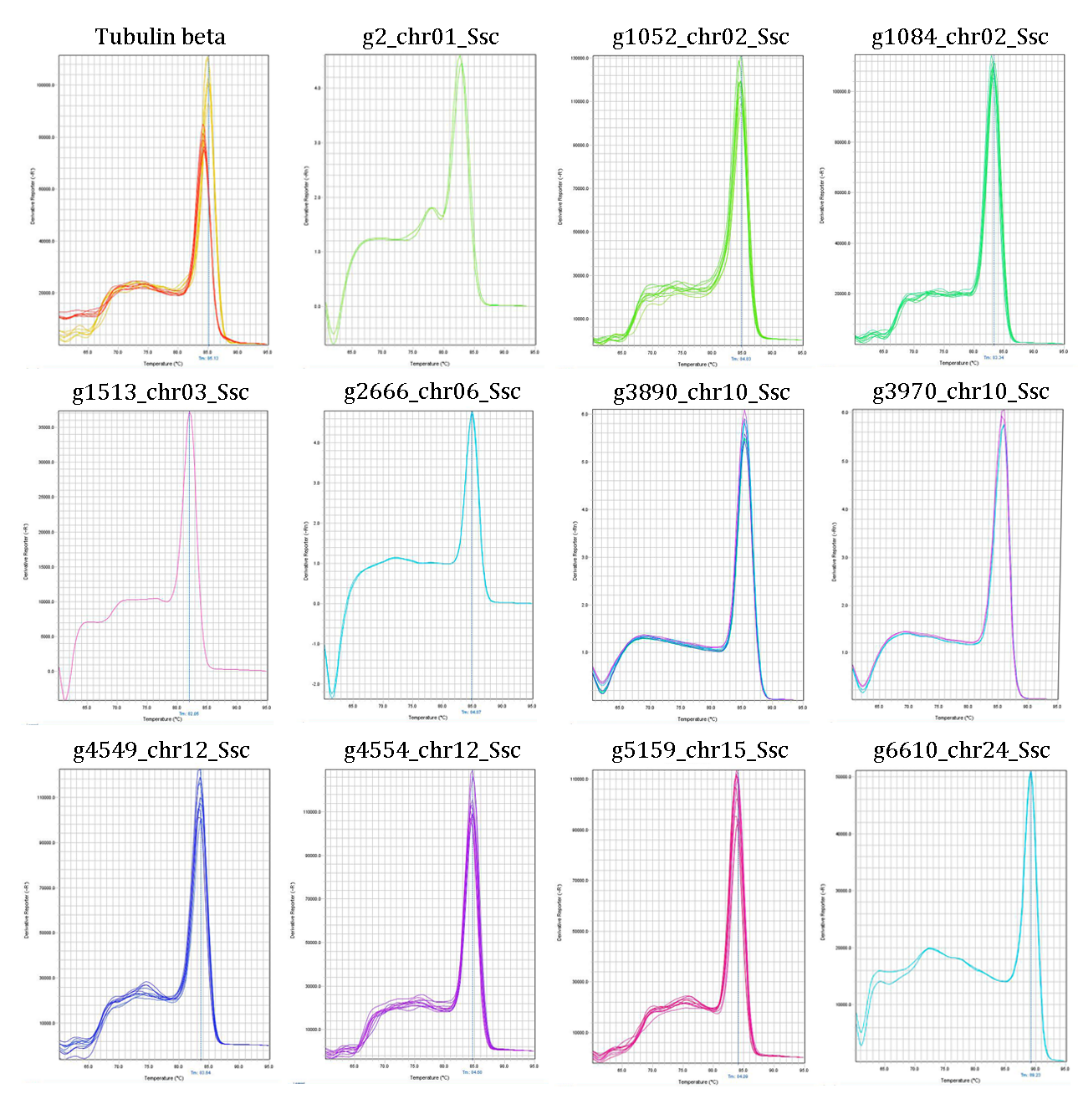


**Figure S4.** **Dissociation curve generated by RT-qPCR reactions for endogenous control and target genes.** Single peaks represent high-quality primers.


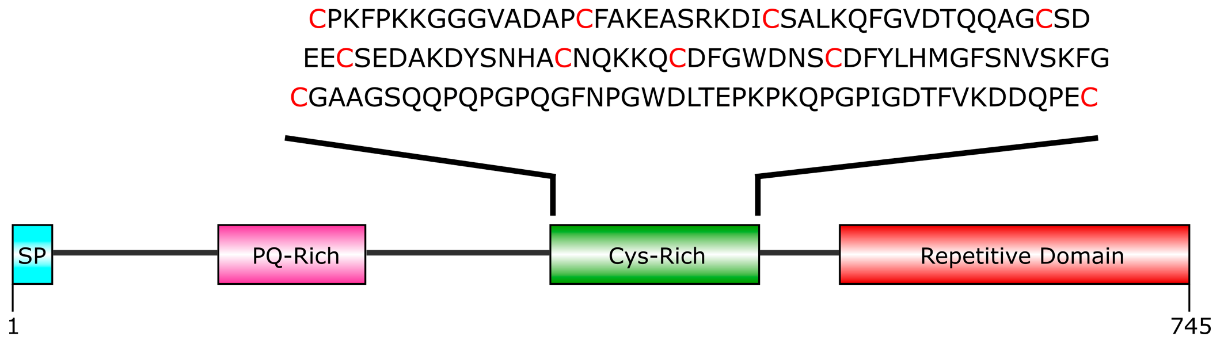


**Figure S5. Protein architecture of g3970.** Diagram shows the signal peptide (SP), the glutamine-proline rich region (PQ-rich), a cysteine-rich region (residues in red color) and the repetitive C-terminal domain.


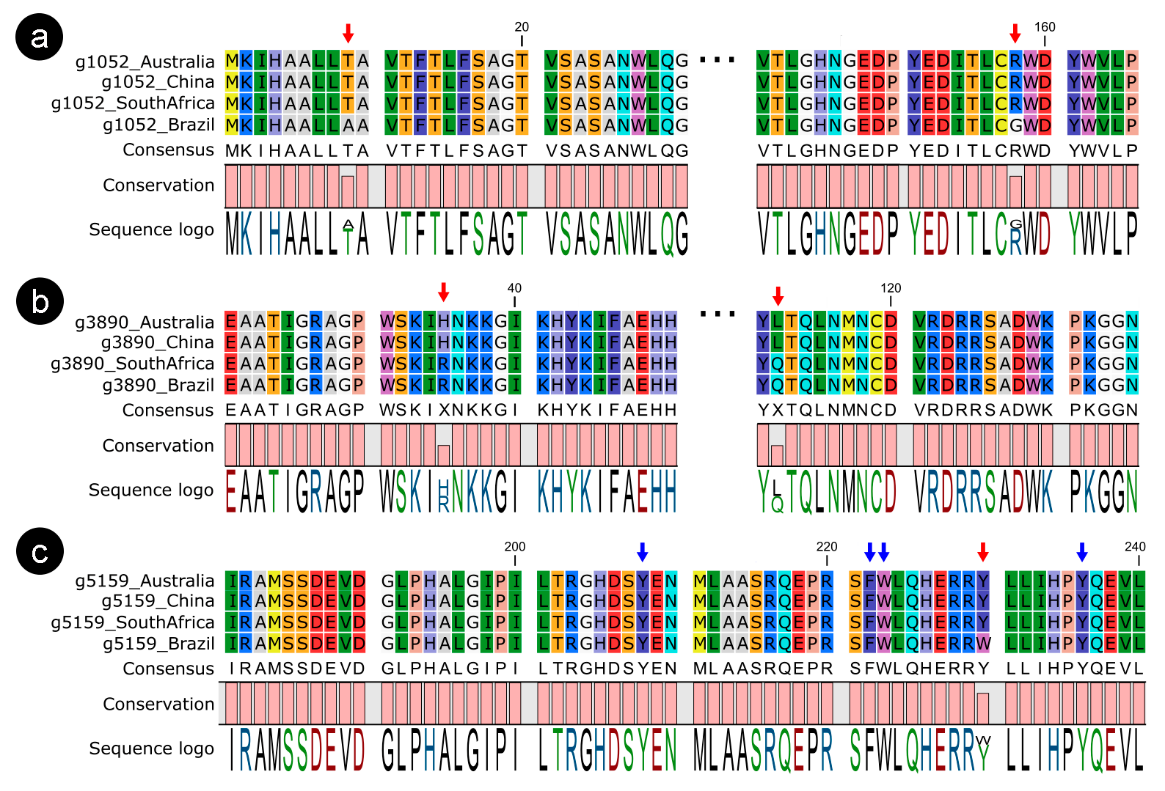


**Figure S6.** **Sequence alignment of candidate effector orthologs from four different strains.** The consensus sequence, and sequence logo showing the polymorphic loci at positions 9 and 158 in g1052 **(a)**, 35 and 112 in g3890 **(b**) and 230 in g5159 **(c)** (red arrows). Note that g5159 polymorphic surrounding region is rich in aromatic residues (blue arrows).


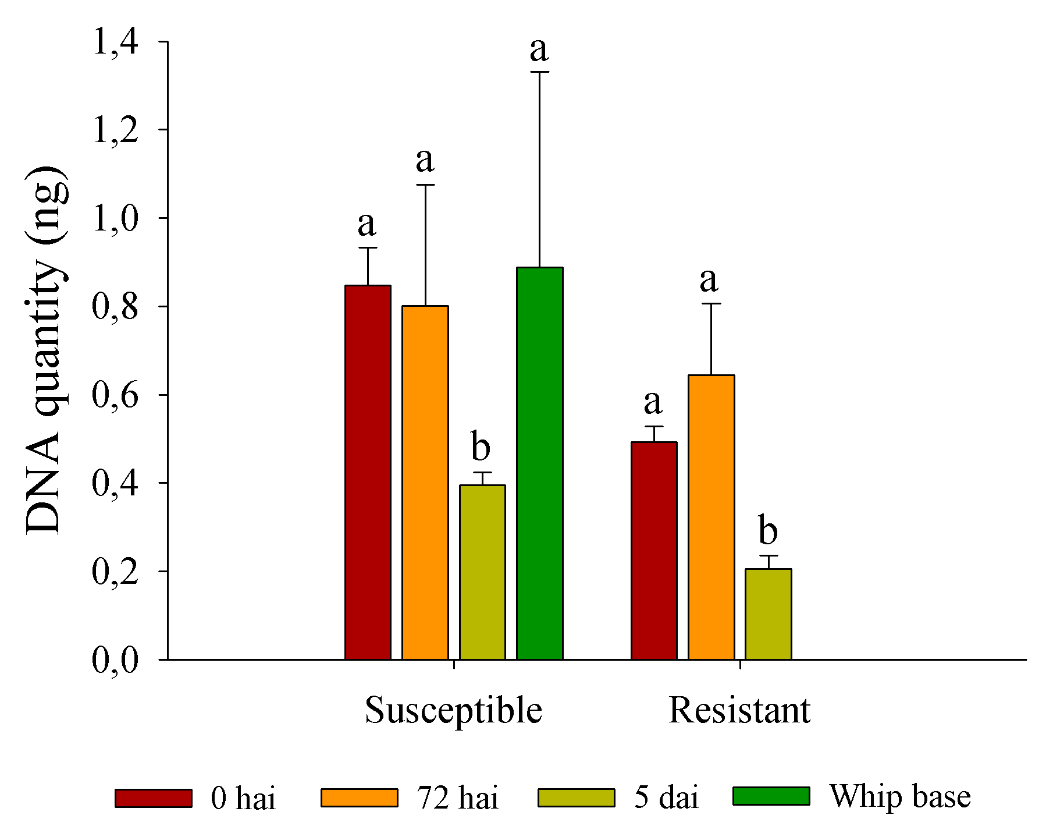


**Figure S7. *S. scitamineum in planta* quantification by qPCR.** Samples were quantified from meristematic tissues of infected plants from two contrasting sugarcane varieties, IAC66-6 (smut susceptible) and SP80-3280 (smut-resistant). Bars represent the amount of pathogen DNA in 100 ng total DNA. Statistical analyses were performed inside each variety. Identical letters represent equivalent means. Error bars indicate standard deviations of three independent biological replicates. dai (days after inoculation).


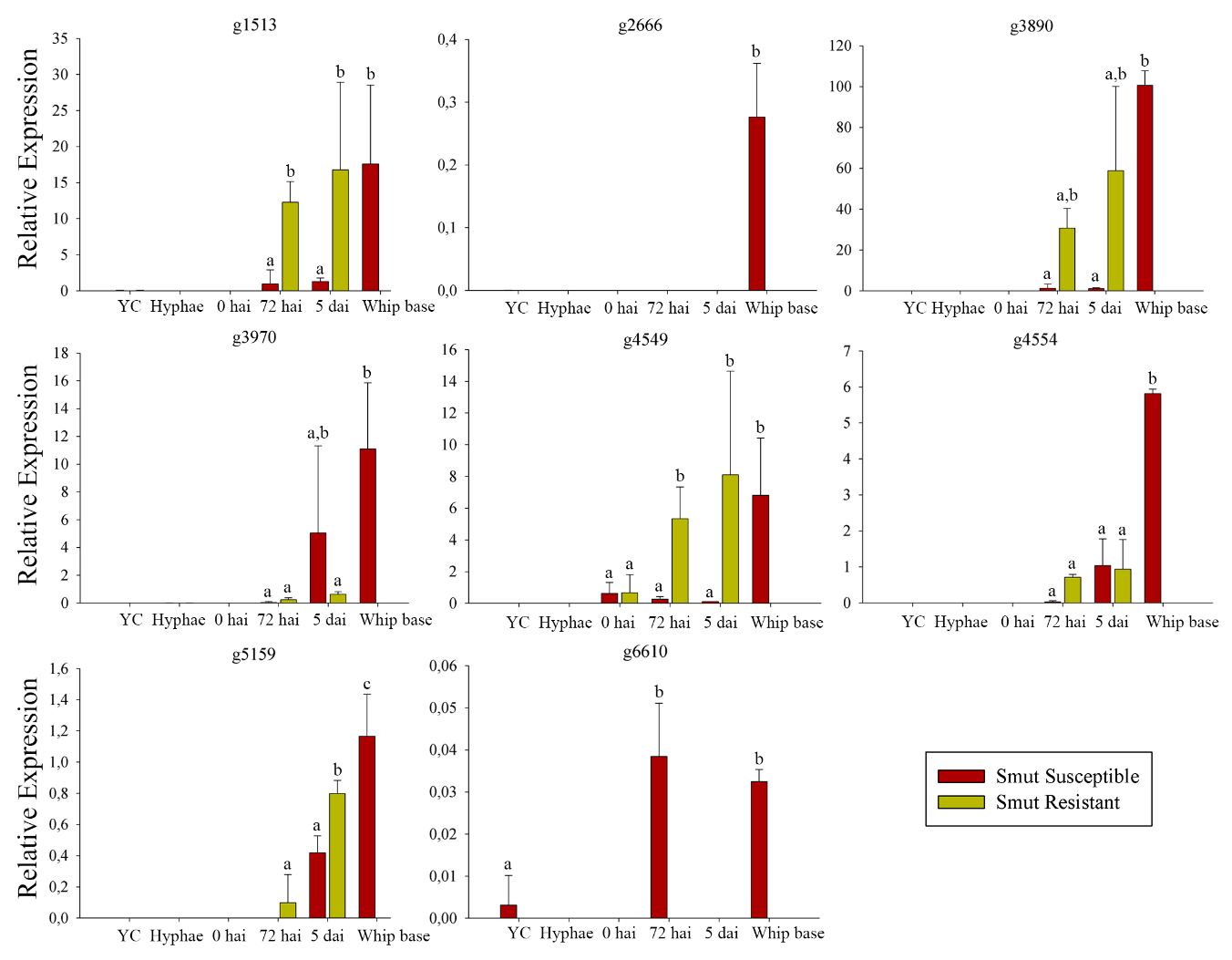


**Figure S8**. ***In planta* expression profile of genes encoding *S. scitamineum* effector candidates by RT-qPCR analysis.** The ΔCt method was used for relative expression calculations. Different letters show significant differences at *P* < 0.05 after a one-way ANOVA followed by Tukey’s test. Error bars represent the standard deviation of three biological replicates. YC (yeast-like cells); dai (days after inoculation).


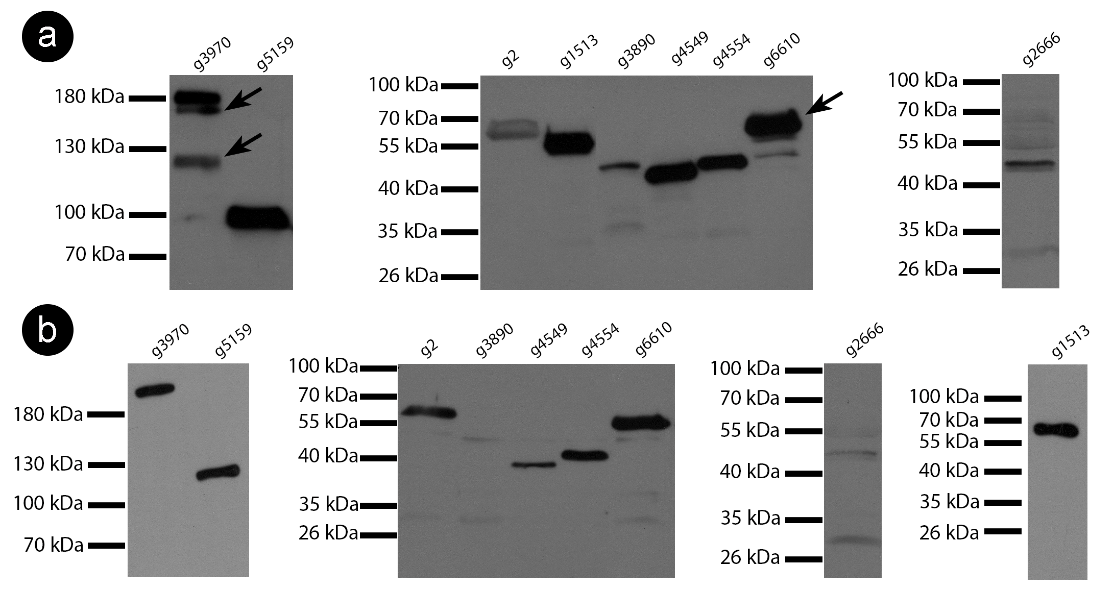


**Figure S9. Immunoblots prepared with total protein extracts.** *S. scitamineum* candidate effector fusion proteins isolated from *N. benthamiana* leaves 3 days after agroinfiltration. Each group represents samples run in the same gel and revealed in the same membrane. **(a)** Citrine C-terminal fusions and **(b)** YFP N-terminal fusions. detected using anti-GFP primary antibody. Arrows indicate additional unexpected bands.


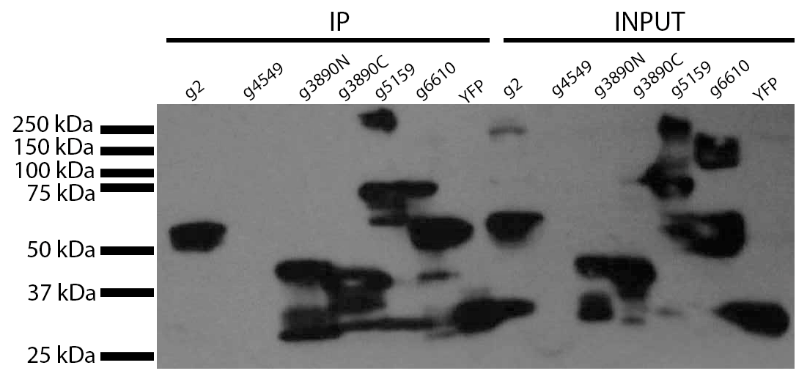


**Figure S10****. Immunoblot prepared with effector fusion proteins after immunoprecipitation with anti-GFP beads.** Samples derived from *S. scitamineum* candidate effector fusion proteins isolated from *N. benthamiana* leaves 3 days after agroinfiltration.


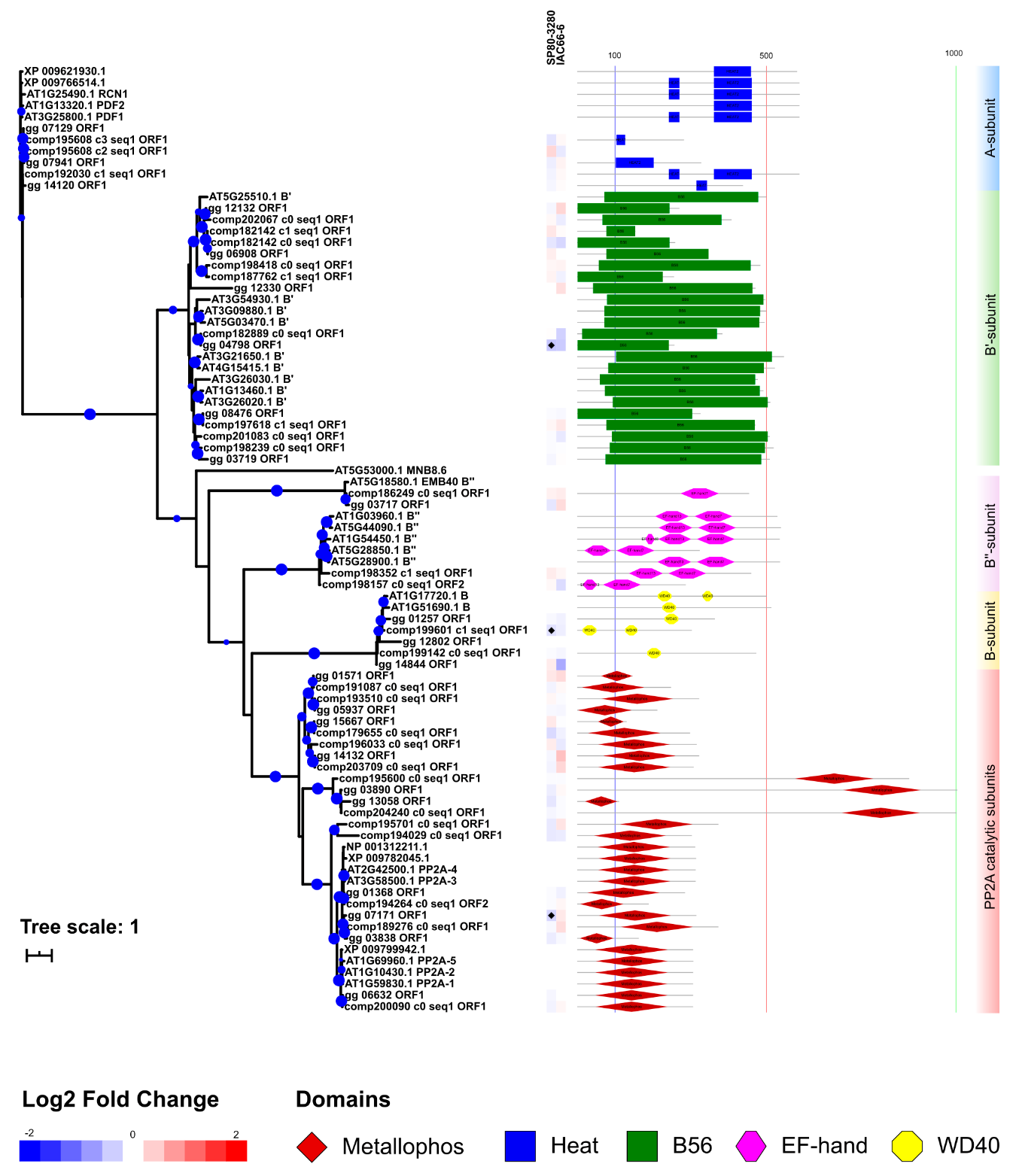


**Figure S11.** **Maximum-Likelihood phylogenetic tree showing the relationships among PP2A orthologs predicted within sugarcane, *Nicotiana*, and *Arabidopsis thaliana* genomes.** Tree was obtained with IQtree software and the JTT+G4 best-fit model of amino acid evolution. Blue circles in tree branches indicate posterior probabilities (PP%) above 75. Expression profiles from sugarcane genotypes of SP80–3280 (smut-resistant) and IAC66–6 (smut-susceptible) are represented as Log2 Fold Change values (inoculated/control) in the heatmap. In the heatmap, blue squares represent down-regulation, whereas red squares represent up-regulation. The statistical significance of expression is depicted by black diamonds if *P* < 0.05. Predicted PFAM protein domains are depicted with colors together with geometric forms according to legend. Dashed grey lines indicate the amino acid sequence length.


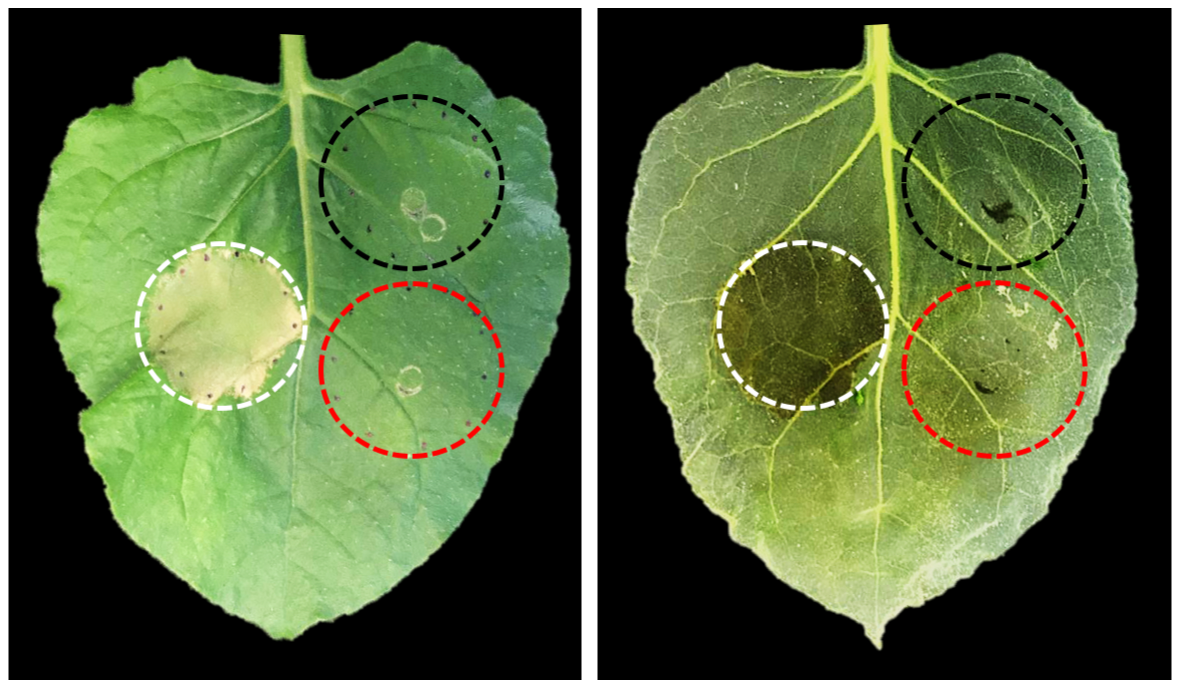


**Figure S12. AvrB activates effector-triggered immunity (ETI) in *Nicotiana benthamiana*.** Leaves (4 to 5 weeks old) were infiltrated with *Pseudomonas fluorescens* EtHAn expressing pVSP6:*:AvrB* (white dotted circle), *P. fluorescens* EtHAn carrying pEDV6 empty vector (black dotted circle), and *P. fluorescens* EtHAn non-transformed (red dotted circle) suspensions at 2 × 10^7^ CFU ml^-1^. The hypersensitive response (HR) was scored at 48 h after infiltrations. Picture shows *N. benthamiana* leaf before (left panel) and after (right panel) decolorization by alcohol bleaching. Photographs were taken at 72 h after co-infiltrations.

| 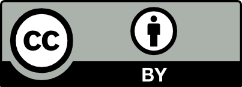 | © 2020 by the authors. Submitted for possible open access publication under the terms and conditions of the Creative Commons Attribution (CC BY) license (http://creativecommons.org/licenses/by/4.0/). |
| --- | --- |
